# Understanding the Effects of Constraint and Predictability in ERP

**DOI:** 10.1162/nol_a_00094

**Published:** 2023-04-11

**Authors:** Kate Stone, Bruno Nicenboim, Shravan Vasishth, Frank Rösler

**Affiliations:** Department of Psychology, University of Potsdam, Potsdam, Germany; Department of Cognitive Science and Artificial Intelligence, Tilburg University, Tilburg, Netherlands; Department of Linguistics, University of Potsdam, Potsdam, Germany; Department of Biological Psychology and Neuropsychology, University of Hamburg, Hamburg, Germany

**Keywords:** N400, anterior PNP, posterior P600, probabilistic processing, constraint, predictability, entropy

## Abstract

Intuitively, strongly constraining contexts should lead to stronger probabilistic representations of sentences in memory. Encountering unexpected words could therefore be expected to trigger costlier shifts in these representations than expected words. However, psycholinguistic measures commonly used to study probabilistic processing, such as the N400 event-related potential (ERP) component, are sensitive to word predictability but not to contextual constraint. Some research suggests that constraint-related processing cost may be measurable via an ERP positivity following the N400, known as the anterior post-N400 positivity (PNP). The PNP is argued to reflect update of a sentence representation and to be distinct from the posterior P600, which reflects conflict detection and reanalysis. However, constraint-related PNP findings are inconsistent. We sought to conceptually replicate Federmeier et al. (2007) and Kuperberg et al. (2020), who observed that the PNP, but not the N400 or the P600, was affected by constraint at unexpected but plausible words. Using a pre-registered design and statistical approach maximising power, we demonstrated a dissociated effect of predictability and constraint: strong evidence for predictability but not constraint in the N400 window, and strong evidence for constraint but not predictability in the later window. However, the constraint effect was consistent with a P600 and not a PNP, suggesting increased conflict between a strong representation and unexpected input rather than greater update of the representation. We conclude that either a simple strong/weak constraint design is not always sufficient to elicit the PNP, or that previous PNP constraint findings could be an artifact of smaller sample size.

## INTRODUCTION

Readers can use contextual cues from words and sentences to construct a mental representation of an event. This representation can be viewed as probabilistic, with plausible upcoming words and sentence structures preactivated in anticipation of their appearance (Kuperberg et al., 2020; Kuperberg & Jaeger, 2016; Kutas & Federmeier, 2011). Assuming that readers generate such a representation, its probabilistic strength should depend on how constraining the sentential context is. For example, in sentence (1)a, the strong constraint of the context makes the word *true* highly predictable, whereas in (1)b, the weak contextual constraint means no specific word is predictable (Federmeier et al., 2007):

(1) 
  - *Strongly constraining*:
    Sam could not believe her story was … true/published
  - *Weakly constraining*:
    I was impressed by how much he … knew/published

The reader’s probabilistic representation should therefore be stronger in (1)a than (1)b, so that encountering the low-predictable word *published* is more unexpected (in the sense that the reader expected a different event) in (1)a, even though *published* is equally unpredictable in both contexts (according to a cloze test; Federmeier et al., 2007). Nonetheless, psycholinguistic measures typically used to study probabilistic processing—including the N400 event-related potential (ERP) component—have been found to correspond only to the matched predictability of *published* between (1)a and (1)b, and not the mismatch in constraint (Federmeier et al., 2007; Kuperberg et al., 2020; Kutas & Hillyard, 1984; Van Petten & Luka, 2012). Instead, an anteriorly distributed positive deflection in the ERP after the N400, the post-N400 positivity (PNP), may hold the key to measuring the constraint/predictability dissociation (Brothers et al., 2020; Federmeier et al., 2007; Kuperberg et al., 2020). However, empirical findings involving the PNP are inconsistent (Federmeier & Kutas, 1999; Frank et al., 2015; Lai et al., 2021; Szewczyk & Schriefers, 2013; Thornhill & Van Petten, 2012; Wlotko & Federmeier, 2007). Given the potential importance of the PNP in studying reader’s probabilistic representations, in this registered report, we addressed possible sample size concerns in previous studies by testing the PNP in a confirmatory study with a larger sample size.

### The Post-N400 Positivity

An incidental finding in many studies of the N400 has been that of a late positivity beginning at around 600 ms in the anterior scalp region. This anterior positivity appears to be spatially and functionally distinct from the more well-known posterior P600 (Kuperberg et al., 2020). The P600 has been variously linked to conflict detection and repair processes in a fronto-temporal cortical circuit (Bornkessel-Schlesewsky & Schlesewsky, 2008; Brouwer et al., 2017; Brouwer & Hoeks, 2013; Fitz & Chang, 2019; Kim & Osterhout, 2005; Kuperberg et al., 2003; Meerendonk et al., 2009; Metzner et al., 2017; Osterhout & Holcomb, 1992). In contrast, the anterior PNP has been linked to the update of event representations, possibly involving the inhibition of representations falsified by unexpected input via left prefrontal cortex (Kutas, 1993). Extending this characterisation, recent research has suggested that the PNP is only elicited when unexpected input is still plausible in the given context (DeLong et al., 2014; Kuperberg et al., 2020). For example, in (2) below, *swimmers* is the most expected continuation, while *trainees* and *drawer* are both low probability. However, *trainees* is still plausible in the context, while *drawer* is not. A PNP and P600 were elicited by *trainees* relative to the expected *swimmers*, but not by *drawer*, which only elicited a P600 (Kuperberg et al., 2020):

(2)  The lifeguards received a report of sharks right near the beach […] Hence they cautioned the swimmers/trainees/drawer

The fact that only the plausible *trainees* and not the implausible *drawer* elicited the PNP has led some to hypothesise that the PNP reflects a change in activity associated with *successfully* updating the mental representation of an event, which may include the inhibition of previous representations (Kuperberg et al., 2020; Kutas, 1993; Ness & Meltzer-Asscher, 2018). Under this assumption and the assumption that the P600 reflects reanalysis (Kim & Osterhout, 2005; Kuperberg et al., 2003; Osterhout & Holcomb, 1992, cf. Bornkessel-Schlesewsky & Schlesewsky, 2008; Brouwer et al., 2017; Fitz & Chang, 2019), Kuperberg et al. (2020) have proposed that an unexpected word (in this example *trainees*) triggers a large but successful update of the readers’ representation of the event, including suppression of the more predictable event *caution the swimmers*. The magnitude of this update is reflected by the presence of a PNP. According to Kuperberg et al. (2020), the unexpected word also engages reanalysis processes during attempts to accommodate it, which are reflected in the presence of a P600. In contrast, the implausible *drawer* triggers no change in the existing event representation (PNP absent), even though reanalysis processes may be engaged (P600 present).

More importantly for research on probabilistic processing, the PNP also appears to be sensitive to contextual constraint. Like the N400, the PNP has been found to be larger for low versus high probability words (Brothers et al., 2017; Brothers et al., 2020; DeLong et al., 2011; DeLong et al., 2014; Federmeier et al., 2007; Kuperberg et al., 2020; Ness & Meltzer-Asscher, 2018; Thornhill & Van Petten, 2012); but unlike the N400, the PNP appears to be larger for low probability words in strongly versus weakly constraining contexts (Brothers et al., 2020; Federmeier et al., 2007; Kuperberg et al., 2020). Returning to the example in (1) above, Federmeier et al. (2007) found that the unexpected word *published* elicited a larger PNP in the strongly constraining (1)a than in the weakly constraining (1)b, even though their cloze probabilities and corresponding N400 amplitudes were the same. The PNP would therefore appear to suggest that a stronger probabilistic representation was built in (1)a than in (1)b, and that the stronger representation was more costly to update.

However, not all studies eliciting the PNP involve a constraint manipulation (Van Petten & Luka, 2012), and thus it is difficult to attribute the PNP exclusively to the manipulation of contextual constraint, rather than to part of a biphasic response to low probability words following the N400. Furthermore, not all studies manipulating constraint show consistent effects on the PNP. Contrary to Federmeier et al. (2007) and Kuperberg et al. (2020), Federmeier and Kutas (1999) found that *expected* words elicited a larger PNP than unexpected words, and only in low constraint sentences. It should be noted that expected words in the Federmeier and Kutas (1999) “low” constraint condition had a mean cloze probability of 0.59 with a range 0.17 to 0.78; nonetheless, the direction of the PNP constraint effect was the opposite of that described elsewhere. In high constraint sentences, no difference in the PNP was observed between expected and unexpected words. More recently, Szewczyk and Schriefers (2013) noted a larger, centrally distributed post-N400 positivity for unexpected versus expected words, but in both high- and low-constraint contexts. Moreover, the effect was found in only two of four conditions involving unexpected words, despite all unexpected words being plausible.

Not only is there inconsistency in how constraint affects the PNP, sometimes constraint-based effects are not elicited at all. In an experiment using the same materials as Federmeier et al. (2007), Wlotko and Federmeier (2007) did not find any evidence of an effect of constraint on the PNP. The lack of a constraint effect on the PNP was perhaps particularly surprising given that constraint was found to affect the earlier P2 component. This dissociation is interesting given that early and late positivities may share a neural generative process, although this is the subject of much debate (Coulson et al., 1998; Osterhout, 1999; Osterhout et al., 1996; Sassenhagen & Fiebach, 2019). If the PNP does indeed share a generative process with the P2, it is therefore surprising that the effect of constraint was not observed in both.

In a study more specifically investigating the PNP, Thornhill and Van Petten (2012) also failed to find any constraint-related difference in PNP amplitude. The authors raise the possibility that the concept of “weak expectation” may need close attention in designing low-constraint experimental stimuli. Low constraint is typically measured using cloze probability; however, the authors suggest that low cloze probability may sometimes reflect a lack of agreement between cloze test participants on the best way to continue a sentence, rather than a “weak” mental representation of the event. More recently, it has been suggested that the *richness* of the mental representation may also determine whether the PNP is seen at an unexpected word (Brothers et al., 2020). For example, in (3)a below, expectation for the upcoming word can only be derived from the three words immediately preceding it. In contrast, in (3)b, a richer context is built across the whole of the preceding sentence. A constraint effect on the PNP was only seen at the unexpected word in (3)b and not in (3)a, suggesting that the richer context allowed a more committed event representation in (3)b, which required a greater update in order to accommodate the unexpected word (Brothers et al., 2020):

(3) 
  - *Locally constraining*:
    He was thinking about what needed to be done on his way home. He finally arrived. James unlocked the door/laptop
  - *Globally constraining*:
    Tim really enjoyed baking apple pie for his family. He had just finished mixing the ingredients for the crust. To proceed, he flattened the dough/foil

One possible explanation for the inconsistency among studies observing a PNP is that its temporal proximity to the N400 makes it susceptible to component overlap (DeLong et al., 2011; Luck, 2005a). Depending on the study design, this may mean that a difference in the PNP is simply the result of an earlier difference in the N400. Other explanations for the inconsistency are that the PNP is simply a broadly distributed P600, or even a methodological artifact. One further complication is that the PNP may have a relationship with the P3 family of components which is as yet unclear (Coulson et al., 1998; Garnsey, 1993; Kuperberg et al., 2020; Kutas & Hillyard, 1980; Osterhout, 1999; Osterhout et al., 1996; Sassenhagen & Fiebach, 2019; Van Petten & Luka, 2012). With these issues in mind, in the present study we treat the N400 and PNP—with temporal and spatial signatures defined by previous research—as distinct measures that can be used to disentangle the influence of contextual constraint. Crucially, the PNP effect should be manipulated by constraint while the N400 should not. Even if the N400 and PNP do arise from generators that exhibit variable latency, finding evidence that they are affected differentially by constraint will still allow conclusions about the usefulness of the PNP in investigating readers’ probabilistic representations. On the other hand, variable latency may obscure any true effect and we may find no support for our hypotheses. In this case, a null result would provide a starting point for future designs or analyses to more explicitly address the contribution of latency variation. With this in mind, we make no claims about the possibility of component overlap or latency variation with respect to the current study.

To summarise, while there is evidence to suggest that the PNP may be sensitive to the strength of readers’ probabilistic sentence representations, there is still inconsistency within the PNP literature. The operationalisation of contextual constraint may also require more careful consideration. Providing strong evidence for an association between the PNP and contextual constraint, and thus a link between the PNP and representation strength, would provide a crucial tool for future research into understanding how probabilistic representations are built, and how readers’ expectations about the upcoming sentence influences their processing of incoming language input.

Moreover, providing further evidence for the PNP establishes a basis with which to investigate the neurobiology of post-N400 positive deflections, including the P600. For example, the link between the PNP and “suppression” (Kuperberg et al., 2020) or “inhibition” (Kutas, 1993; Ness & Meltzer-Asscher, 2018) suggests engagement of executive processes in the prefrontal cortex (e.g., Hagoort, 2013). These executive processes are proposed to have a distinct cortical location and function from the types of processes to which the P600 is sensitive (Hagoort, 2013; Hagoort & Indefrey, 2014). The P600 is instead proposed to index involvement of circuits between the left inferior prefrontal cortex and the temporal lobe as information from memory is retrieved and integrated during attempts to revise a disconfirmed sentence representation (Brouwer et al., 2017; Brouwer & Hoeks, 2013). Strong evidence for the PNP would aid future investigations in this direction.

### The Current Study

Recent research efforts have highlighted the fact that one of the critical findings in research on probabilistic preactivation is difficult to replicate (Nieuwland et al., 2018) and that the effect sizes of this predictability manipulation is likely much smaller than thought (Nicenboim et al., 2020). Overestimated effect sizes and/or effects in an unexpected direction can be the result of Type M(agnitude) and S(ign) errors in underpowered study designs with too few participants and/or too few experimental items (Gelman & Carlin, 2014). ERP experiments are particularly susceptible to being underpowered given that they are costly, both in terms of time, labour, equipment maintenance, and replacement of disposable elements. Resource constraints therefore may prevent the recruitment of a sufficient number of participants to offset the high level of signal-to-noise ratio inherent in ERP data (Luck, 2005a; Luck & Gaspelin, 2016). Many ERP studies also involve the comparison of ERP components at target words that are not identical, which may introduce additional noise through variability in frequency and lexical representations. Investigation of the PNP would therefore greatly benefit from a confirmatory study using a large number of participants.

We expected to show a dissociated effect of constraint on the N400 and PNP in a relatively large number of participants (see Participants below). The key findings that we wished to replicate were those of Federmeier et al. (2007) and Kuperberg et al. (2020), who found that only the PNP and not the N400 was affected by constraint. We extended the design of Federmeier et al. by measuring PNP and N400 effects at matching words with matching pre-critical regions, eliminating any potential lexical- or frequency-based variation. Kuperberg et al. (2020) also measured ERPs at matching words, but we extended their design by operationalising contextual constraint as the continuous variable “entropy.” Entropy is a measure of uncertainty at the target word that takes into account how the context of a sentence has affected the distribution of probable words at that position (see Cloze test below for a more detailed definition). In addition, we used constraint (entropy) and word predictability (log cloze probability) as continuous rather than categorical predictors in the statistical analysis, which maximises statistical power (Cohen, 1983). A discussion of the use of log cloze probability can be found in Analyses. A successful replication would make a solid contribution to evidence that the PNP will be of great value in future investigations of probabilistic processing.

## MATERIALS AND METHODS

The Introduction and Materials and Methods sections of this manuscript received Stage 1 approval as a registered report and were pre-registered at https://osf.io/bxg3n.

### Participants

In total, electroencephalography (EEG) was recorded from 74 participants. Seven participants were excluded due to software problems during the recording and three because more than 75% of their EEG was affected by artifact. This left a final sample size of 64. The participant sample size was determined via a stopping rule based on the inference criteria used in our statistical analysis (the Bayes factor), as well as time and resource limitations. We planned to recruit participants either until we reached a Bayes factor of 10 in favour of the null or the alternative hypotheses, or until we reached 150 participants, whichever came first. 150 participants was thought to be the maximum feasible number that we could collect data from given limited resources and time. However, a major protocol deviation was made with the approval of the editor and reviewers: A Bayes factor of 10 was exceeded for the PNP constraint effect at 40 participants, but the Bayes factor for the N400 constraint effect remained stable at approximately 1, regardless of sample size. Due to the difficulty in recruiting participants during the COVID-19 pandemic and because it seemed unlikely that the Bayes factor for the N400 constraint effect would reach 10 even with 150 participants, we ceased recruitment early. We discuss the inconclusive Bayes factor further in the Results section and present a design analysis which suggests that even over 150 participants would not have been sufficient to reach the pre-registered Bayes factor threshold.

More detail on the statistical analysis is provided below, but support for our hypotheses was assessed using Bayes factors for the effect of entropy (PNP prior: a truncated normal distribution *N*_−_(0, 0.2); N400 prior: a normal distribution *N*(0, 0.2)), and cloze probability (PNP prior: a truncated normal distribution *N*_−_(0, 0.2); N400 prior: a truncated normal distribution *N*_+_(0, 0.2)). Statistical Models and Predictions provides further detail and motivates the use of truncated prior distributions.

Even with the protocol deviation, to our knowledge, the sample size is the largest amount of data to date on this topic, and we reached strong evidence (a Bayes factor of at least 10, in line with Jeffreys, 1939) in favour of two pre-registered hypotheses without reaching the maximum of 150 participants. For the hypotheses for which even 150 participants would not have yielded strong evidence, the experiment is still informative because the estimates from our data can be used in a future meta-analysis in order to synthesise the evidence available so far. For examples illustrating the importance of evidence synthesis in psycholinguistics, see Bürki et al. (2022), Jäger et al. (2017), Nicenboim et al. (2020), and Vasishth and Engelmann (2021).

The inclusion criteria for participants in the study were: native German speakers with no other language acquired before age 6, no history of developmental or acquired reading, production, or hearing disorder, no history of developmental or acquired neurological disorder, and no current need for or intake of psychopharmaceutical medication. All participants’ vision was normal or corrected to normal. Participants were excluded from the final analysis if there were technical problems with the EEG recording, if more than 75% of EEG segments were badly affected by artifact, or if the attention check was failed (post-stimulus questions answered with an accuracy of less than 70%).

### Materials

Each experimental item consisted of four sentences. An example item is below. In the example, target nouns for the respective analyses are in bold face:

(4) 
  - *Strong constraint, high cloze probability noun*:
    Auf Annetts Terrasse schien im Sommer zu viel Sonne, um noch draußen sitzen
    On Annett’s terrace shone in summer too much sun in order outside sit
    zu können. Daher kaufte sie sich einen großen **Schirm** und …
    to be able. Therefore bought she herself a._MASC_ large._MASC_ **umbrella.**_**MASC**_ and …
  - *Strong constraint, low cloze probability noun*:
    Auf Annetts Terrasse schien im Sommer zu viel Sonne, um noch draußen sitzen
    On Annett’s terrace shone in summer too much sun in order outside sit
    zu können. Daher kaufte sie sich einen großen **Hut** und …
    to be able. Therefore bought she herself a._MASC_ large._MASC_ **hat.**_**MASC**_ and …
  - *Weak constraint, low cloze probability noun*:
    Annett mag es gerne gemütlich, wenn sie etwas Zeit für sich findet. Daher
    Annett likes it really cozy when she some time for herself finds. Therefore
    aufte sie sich einen großen **Schirm** und …
    bought she herself a._MASC_ large._MASC_ **umbrella.**_**MASC**_ and …
  - *Weak constraint, low cloze probability noun*:
    Annett mag es gerne gemütlich, wenn sie etwas Zeit für sich findet. Daher
    Annett likes it really cozy when she some time for herself finds. Therefore
    kaufte sie sich einen großen **Hut** und …
    bought she herself a._MASC_ large._MASC_ **hat.**_**MASC**_ and …

#### Cloze test

To assess noun predictability, native German speakers completed sentences truncated after the determiner before the target noun. For the strongly constraining conditions, we used the publicly available stimuli from Nicenboim et al. (2020) and so the cloze procedure for the strongly constraining condition is as reported in that paper. For the weakly constraining condition, 60 new participants completed truncated sentences presented in Ibex (Drummond, 2016), either in the lab or online via Prolific (www.prolific.co). Plural and singular forms of the same word were collapsed, as were nouns with the same stem (e.g., *Schirm* “umbrella” and *Sonnenschirm* “sun umbrella” or “parasol”). The cloze probability of the target noun in each condition was computed as the proportion of participants who gave that word or word stem out of the total number of participants.

To assess the contextual constraint of our conditions, we calculated entropy at the noun site. Entropy is a measure of uncertainty in terms of how the probability mass of cloze test responses is distributed. For example, in a strong constraint context, nine cloze test completions may be the word “umbrella” and one may be “hat.” Probability mass is therefore concentrated on “umbrella” and entropy is low (high constraint). In a weak constraint context, the cloze completions may be 10 different words; now probability mass is evenly distributed and entropy is high (low constraint). We quantified Entropy (H) as the negative sum of cloze probabilities (P) for all nouns provided by participants for a particular sentence in the cloze test, multiplied by their respective logs: *H* = −$\sum_{i=1}^{n}$ *P*_*i*_ log *P*_*i*_. For example, if nine cloze completions were “umbrella” and one was “hat” then: *H* = −(*P*_*umbrella*_ · log *P*_*umbrella*_ + *P*_*hat*_ · log *P*_*hat*_) = −(0.9 · log 0.9 + 0.1 · log 0.1) = 0.47. Summary statistics for cloze probability and entropy are reported in Table 1 as well as in Appendix B, Figure B1, in the Supporting Information, available at https://doi.org/10.1162/nol_a_00094.

**Table 1.** Cloze probability and entropy descriptive statistics.

Condition	log_2_ cloze probability		Proportion target word		Entropy (bits)	
	Mean	95% range	Mean	95% range	Mean	95% range
a) Strong constraint,high predictable noun	−0.40	−1.00, −0.07	79.60	50.00, 100.00	0.68	0.00, 1.59
b) Strong constraint, low predictable noun	−3.71	−4.58, −2.50	5.47	4.17, 14.60	0.68	0.00, 1.59
c) Weak constraint, low predictable noun	−4.09	−5.09, −1.51	7.49	2.94, 34.20	2.44	1.47, 3.12
d) Weak constraint, low predictable noun	−4.46	−5.09, −2.34	4.93	2.94, 17.80	2.44	1.47, 3.12

*Note*. log_2_ cloze probability is presented, as log_2_ cloze probability was used in the statistical model. Since cloze probability can only range between zero and one, log_2_ cloze probability values ranged between minus infinity and zero. The 95% range refers to the 2.5th and 97.5th percentiles of the data. Proportion target word refers to the raw percentage of cloze completions where the target word was given. Entropy reflects contextual constraint, where low values indicate strong constraint (low variety of completions given), and high values weak constraint (high variety of low probability completions given).

### Design

Sentences were constructed in quartets, although the experimental design was nonfactorial, with conditions (a) and (b), and (b) and (d) being collapsed in two respective analyses. Condition (c) was presented for lexical balance:

- *Strong constraint, high predictable noun*
- *Strong constraint, low predictable noun*
- *Weak constraint, low predictable noun*
- *Weak constraint, low predictable noun*

Stimuli were presented in a Latin square design such that all participants saw only one sentence from each item. There were 224 items in total. The collapsed conditions meant that in each analysis, each participant would contribute data from 112 items. Since all sentences were grammatical and plausible, filler sentences were not used.

### Procedure

Participants were tested in a single session. For the EEG recording, participants were seated in a shielded EEG cabin at distance of approximately 60 cm from a 56 cm presentation screen. The experimental presentation paradigm was built using OpenSesame (Mathôt et al., 2012). Each experimental session began with instruction screens advising participants that they would read two related sentences for each trial: The first sentence was presented several words at a time and the second (the critical sentence) was presented word-by-word. Participants were advised that after some sentences, they must answer a question as quickly and accurately as possible. Each experimental session began with five practice trials.

Each trial in the experiment began with a 500 ms fixation cross in the centre of the screen followed by a blank screen jittered with a mean of 1,000 ms and standard deviation of 250 ms. Each sentence was presented word-by-word for a duration of 190 ms per word plus 20 ms for each letter. The target word, however, was presented for 700 ms regardless of length so that the segment of EEG on which we conduct our analysis would not include the onset of the following word. The interstimulus interval was 300 ms. After 50% of the sentences, a yes/no comprehension question appeared; for example, *Hat Annett eine Terrasse?* (Does Annett have a terrace?). Answering the question via a video game controller triggered the beginning of the next trial. The order of presentation of sentences within each list was fully randomised by the presentation software. Breaks were offered after every 30 sentences.

Before starting the EEG experiment, participants performed a stop signal task (Lappin & Eriksen, 1966; Logan & Cowan, 1984) that closely followed the design of Verbruggen et al. (2008). The purpose of the stop signal task was to measure individual differences in the ability to stop an action (a button press) once they had already initiated it. This information was correlated with participants’ PNP responses, with the hypothesis that poorer performance on the stop signal task may correlate with smaller constraint-related differences in the PNP; that is, if the PNP is related to suppressing the mental representation of a sentence that has been falsified by unexpected input, people who are better at inhibiting responses on the stop signal task might also show larger PNP constraint effects. However, this was an exploratory analysis and we pre-registered no specific analysis plan here. The testing session including EEG setup lasted approximately three hours.

### EEG Recording Parameters and Preprocessing Pipeline

EEG was recorded from 32 scalp sites by means of AgAgCl active electrodes mounted in an elastic electrode cap at the standard 10–20 system (Jasper, 1958). Eye movements and blinks were monitored with bipolar electrodes next to the left and right outer canthus as well as below and above the right eye. EEG and electrooculography was recorded with a TMSi Refa amplifier with active shielding at a sampling rate of 512 Hz and a low-pass filter of 138 Hz, in line with manufacturer recommendations. Recordings were initially referenced to the left mastoid and re-referenced offline to the average of the left and right mastoid channels.

EEG was filtered offline using zero phase finite impulse response (FIR) filters with a bandpass of 0.01–30 Hz on whole, unsegmented EEG blocks (i.e., continuous blocks recorded between participants’ breaks). The width of the transition band at the low cut-off frequency was 0.01 Hz and at the high cut-off frequency, 7.5 Hz. Data were then segmented into whole sentences and blinks and eye movements corrected using independent component analysis (ICA; Jung et al., 2001) with the Fast ICA algorithm (Hyvärinen & Oja, 2000). ICA components were inspected for each participant and removed if they strongly correlated with the ocular channels. The data were then further segmented to extract the target region, and segments were rejected if they contained a voltage difference of over 100 *μ*V in a time window of 150 ms or containing a voltage step of over 50 *μ*V/ms. In total, this pipeline resulted in the rejection of 16% of the target noun segments, leaving approximately 3,000 target segments per condition. Corrected signal was then segmented and baseline-corrected relative to a 200 ms interval preceding the stimulus.

### Analyses

The dependent variables in our planned analyses were:

- N400: Average ERP amplitude (*μ*V) over electrodes Cz, CP1, CP2, P3, Pz, P4, and POz in the window 300–500 ms following target word onset.
- PNP: Average ERP amplitude (*μ*V) over electrodes Fpz, Fp1, Fp2, F3, Fz, F4 in the window 600–1000 ms following target word onset.

As mentioned above, constraint was operationalised as entropy, where increasing entropy reflected decreasing constraint. Noun predictability was operationalised as smoothed cloze probability transformed to log_2_. Additive smoothing was used with pseudocounts set to one to avoid taking the log of zero (Laplace or Lidstone smoothing; Chen & Goodman, 1999; Lidstone, 1920). The log transformation reflected the assumption that the effect of cloze probability on N400 amplitude is continuous and nonlinear. In other words, changes in cloze probability at the upper end of the probability scale will not affect N400 amplitude as much as changes at the lower end of the scale. Thus, the model will estimate the same average change in amplitude for a difference in cloze probability of 0.09 to 0.26 as for a change of 0.26 to 0.74, even though the latter represents a larger change in raw cloze probability. Log transformed cloze probability has previously been demonstrated to give a better fit to ERP data (Delaney-Busch et al., 2019; Frank et al., 2015; Nicenboim et al., 2020), as well as to reading time data (Hale, 2001; Levy, 2008; Smith & Levy, 2013), is consistent with Pareto and Zipf distributions of word frequency (Baayen, 2001), and with scaling laws in other areas of cognitive research (Kello et al., 2010).

Both entropy and log cloze probability were centred according to the mean of the conditions included in the model (see below), such that the model estimated the one-unit change in ERP amplitude at average values of log cloze probability and entropy (average values are in Table 1 above).

#### Statistical models and predictions

Linear mixed effects models with correlated by-item intercept estimates and full variance-covariance matrices for by-subject random effects were fit in the rstan/Stan wrapper brms (Version 2.16.3; Bürkner, 2017) in R (R Core Team, 2020). (For a complete list of the software used in this article, see Software, below.) Only random intercepts were estimated for items because once the conditions were collapsed to treat entropy and cloze probability as continuous predictors, there were only two entropy/cloze values per item (corresponding to each sentence context). Since this was unlikely to be sufficient to precisely calculate by-item random slopes, to reduce computation time we included by-item intercepts only.

Our priors for the models were informed by the model estimates of previous Bayesian ERP analyses, which suggested that intercept variability was higher than individual variability between participants and items (Nicenboim et al., 2020). Using prior predictive checks against simulated data, we then calibrated the priors so that they were in line with previous findings, but not strictly informative. These regularising priors were used to ensure stable and psycholinguistically plausible estimates (Chung et al., 2015; Gelman et al., 2008; Gelman et al., 2017). We confirmed that the joint behaviour of these priors in the model would generate plausible estimates using prior predictive checks (Gelman et al., 2017; Schad et al., 2021); see Figure 3 in Prior Distributions and Predictive Check for the Statistical Models. The priors were:

$$\begin{array}{c} \text{intercept}∼\text{Normal}\left(0,5\right)\\ \beta_{\text{predictability}}∼\text{Normal}\left(0,1\right)\\ \beta_{\text{constraint}}∼\text{Normal}\left(0,1\right)\\ \sigma_{\text{subject},\text{item}}∼{\text{Normal}}_{+}\left(0,0.5\right)\\ \sigma_{\text{residual}}∼{\text{Normal}}_{+}\left(8,2\right)\\ \rho ∼LKJ\left(2\right) \end{array}$$

Models for estimation were fit with 50,000 iterations, including a warmup of 1,000 iterations. Model convergence was assessed by ensuring that the number of bulk and tail effective samples for every parameter estimate was at least 2,000 and that $\hat{R}$ values—the correlations of between- and within-chain variance—did not exceed 1.01. If these checks were violated, the number of iterations for each model was increased, or sampler behaviour modified, as indicated by warning messages from brms.

Support for our specific hypotheses (detailed below) was assessed using Bayes factors. As we had very specific, pre-registered hypotheses about the direction of these effects, the priors used for the Bayes factor analysis were truncated such that they constitute one-sided tests. As discussed above, conclusions about evidence for or against our hypotheses was based on Bayes factors computed using priors of *Normal*_−_(0, 0.2) for the effect of entropy (constraint) and cloze probability (predictability) on the PNP, and *Normal*(0, 0.2) for the effect of entropy (constraint) and *Normal*_+_(0, 0.2) for the effect of cloze probability (predictability) on the N400, according to which of the questions (see Statistical Models and Predictions and Prior Distributions and Predictive Check for the Statistical Models) was being tested. These truncated priors were used for hypothesis testing, but exploratory analyses with two-sided tests was also used to assess evidence for non-hypothesised effects.

Models for the Bayes factor analyses were fit with 50,000 iterations in line with Bürkner (2017) recommendations, including a warmup of 1,000 iterations. Convergence was assessed as for the estimation models—at least 2,000 bulk and tail effective samples for each parameter estimate, and $\hat{R}$ ≤ 1.01. Bayes factors were calculated using bridge sampling (Bennett, 1976; Gronau et al., 2017; Meng & Wong, 1996). The strength of evidence for or against our hypotheses was assessed with reference to Jeffreys (1939) scale, where a Bayes factor indicating evidence at a ratio of 3:1 in favour of an effect is considered the minimum meaningful support for that effect, and only 10:1 or larger values are considered strong evidence. Given the sensitivity of the Bayes factor to the choice of prior (Lee & Wagenmakers, 2014), we also computed Bayes factors for a range of different priors on the effects of constraint (entropy) or predictability (cloze probability) while holding all other priors (e.g., intercept, random effects) constant as defined above. The priors for these sensitivity analyses ranged from *Normal*(0, 0.2) to *Normal*(0, 2), both truncated and non-truncated.

##### Effect of low predictability at the noun under differing constraint.

Our main comparison of interest concerned the effect of constraint when noun predictability was low. With respect to the N400, in line with previous research we expected that words with similar cloze probabilities would elicit N400s with similar amplitudes, regardless of how constraining their context was. With respect to the PNP, if it is the case that the PNP reflects the cost of revising a probabilistic event representation (Kuperberg et al., 2020), then we should expect that low cloze probability words elicit a PNP that is larger in contexts that are strongly constraining than in contexts that are weakly constraining.

**Figure 1.**
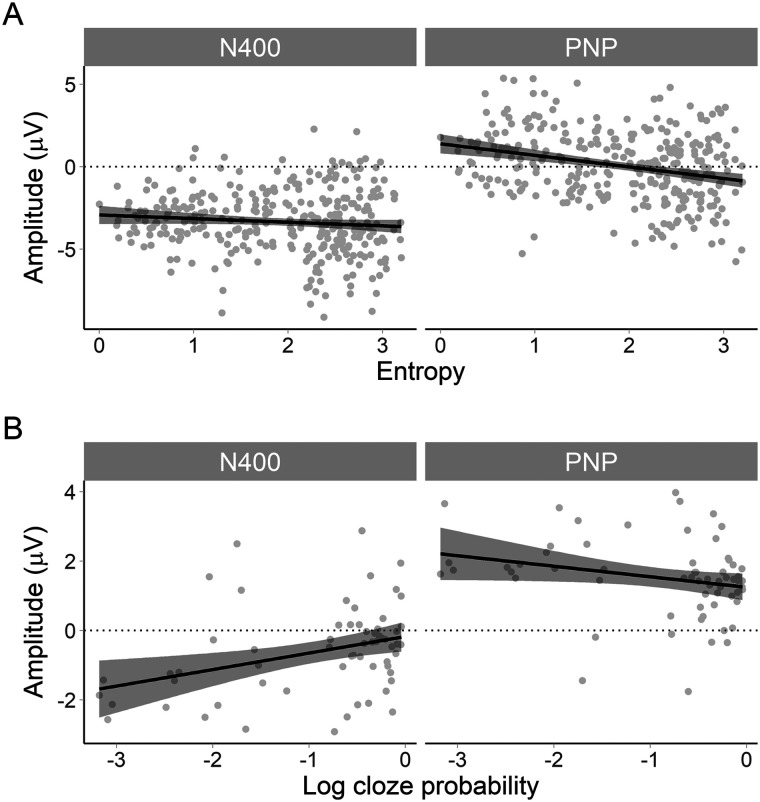
Simulated direction of the effect of constraint and predictability on average amplitude in the N400 and PNP time windows. (A) In our first analysis, we collapsed conditions (b) and (d) such that predictability (cloze probability) was low but constraint (entropy) varied. Increasing entropy means decreasing constraint. Thus, as entropy increases on the *x*-axis, PNP amplitude should become less positive. In other words, the PNP at unexpected words should be more positive at low values of entropy (high constraint) than at high values of entropy (low constraint). N400 amplitude should not be affected by constraint, but may be sensitive to small differences in cloze probability between conditions (b) and (d). This was accounted for in the statistical analysis by adding cloze probability as a nuisance variable. (B) In our second analysis, we collapsed conditions (a) and (b) such that constraint was high (low entropy), but predictability (cloze probability) varied. Cloze probability values are negative due to the log transformation. As cloze probability increases toward zero on the *x*-axis, the N400 becomes less negative and the PNP less positive. In other words, as predictability increases, the size of both the N400 and the PNP decrease.

For this comparison, we took sentences from conditions (b) and (d), which both had low cloze probability nouns but varied in entropy (high entropy = weak constraint, low entropy = strong constraint); this can be seen in Figure 1A. Conditions (b) and (d) were collapsed together and ERP amplitude analysed as a function of continuous entropy. Although noun cloze probability in both conditions was low, there was some variability due to the differing contexts and thus log cloze probability was added as a continuous nuisance predictor in the models. In short, Figure 1A shows our predictions that when cloze probability is low:

- the N400 would be of equally high (negative) amplitude regardless of entropy (constraint). There may be a small effect of cloze probability;
- the PNP would become more positive as entropy decreases (i.e., as constraint increases). There may be a small effect of cloze probability.

Note that cloze probability and entropy are somewhat correlated (see Appendix B, Figure B1). This is because it is difficult to build stimuli that hold cloze probability constant while systematically varying entropy. However, our pre-registered hypotheses do not concern the effect of an interaction, and adding an interaction term to the model may only estimate variance otherwise explained by entropy (or cloze probability). For this reason, we chose to omit an interaction from the model.

R brms model specification:

$$\begin{array}{l} N400∼\text{constraint}+\text{predictability}+\left(1|\text{item}\right)+\left(1+\text{constraint}+\text{predictability}|\text{subj}\right)\\ PNP∼\text{constraint}+\text{predictability}+\left(1|\text{item}\right)+\left(1+\text{constraint}+\text{predictability}|\text{subj}\right) \end{array}$$

##### Effect of differing predictability at the noun under strong constraint.

As a sanity check, we also compared conditions (a) and (b). It is well established that decreasing cloze probability should increase amplitude of the N400 (i.e., make it more negative; Kutas & Federmeier, 2011) and of the PNP (i.e., make it more positive; Federmeier et al., 2007; Kuperberg et al., 2020). Under this assumption, when constraint was matched, we expected a larger N400 and PNP for low versus high cloze probability words. For this comparison, we took sentences from conditions (a) and (b), which both had strong constraint but varied in cloze probability; see Figure 1B. Thus, conditions (a) and (b) were collapsed and ERP amplitude analysed as a function of continuous log cloze probability. As can be seen in Figure 1B, we expected that when constraint was strong:

- the N400 would become more negative as cloze probability decreases;
- the PNP would become more positive as cloze probability decreases.

R brms model specification:

$$\begin{array}{l} N400∼\text{predictability}+\left(1|\text{item}\right)+\left(1+\text{predictability}|\text{subj}\right)\\ PNP∼\text{predictability}+\left(1|\text{item}\right)+\left(1+\text{predictability}|\text{subj}\right) \end{array}$$

#### Prior distributions and predictive check for the statistical models

**Figure 2.**
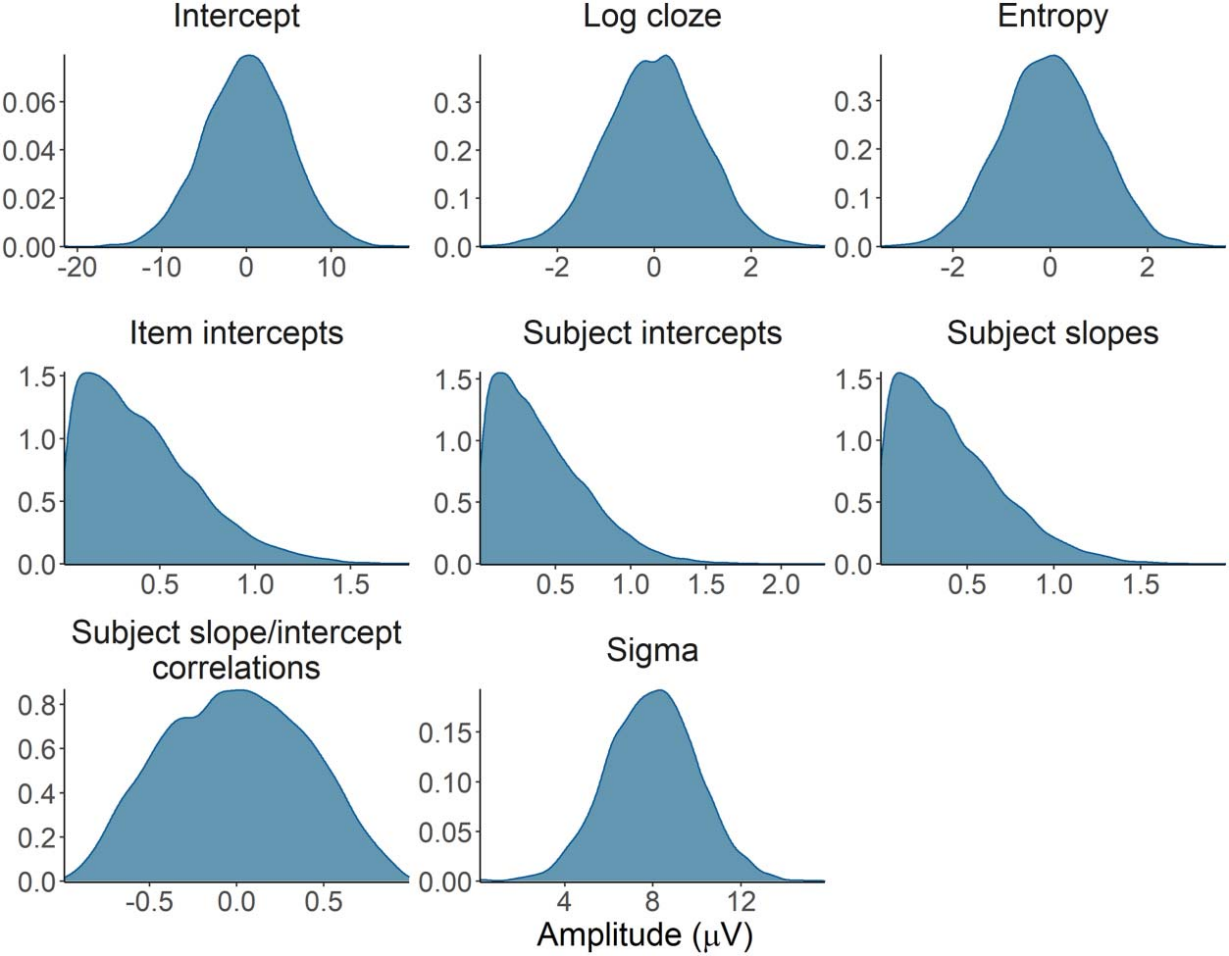
Prior distributions for the model parameters.

**Figure 3.**
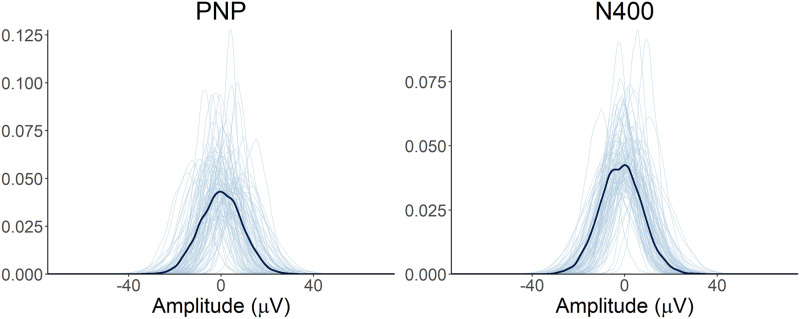
Prior predictive check. Prior predictive distributions for the effect of entropy on the PNP and N400 (light blue lines) based on the model priors suggests the priors generate plausible estimates consistent with simulated data (dark blue lines).

As an additional check that our prior specification would result in sensible estimates for our models, we conducted a prior predictive check (Gelman et al., 2017; Schad et al., 2021). In Figure 2, we show the prior distributions for each parameter in our statistical models. In Figure 3, we show the posterior distributions of a model simulating the predicted effect of entropy on the PNP and the N400 using only the priors. The estimated effect of entropy based on the priors (light blue lines) is plausible with respect to the effect based on simulated data (dark blue line), confirming that the joint behaviour of our priors in the model did not lead to implausible parameter estimates.

## RESULTS

In the following sections we report first the results of the pre-registered analyses, then the results of our exploratory analyses. Data and code for all analyses are available at https://osf.io/fndk5.

### Preregistered Analysis

#### Effect of low predictability at the noun under differing constraint

##### PNP window.

**Figure 4.**
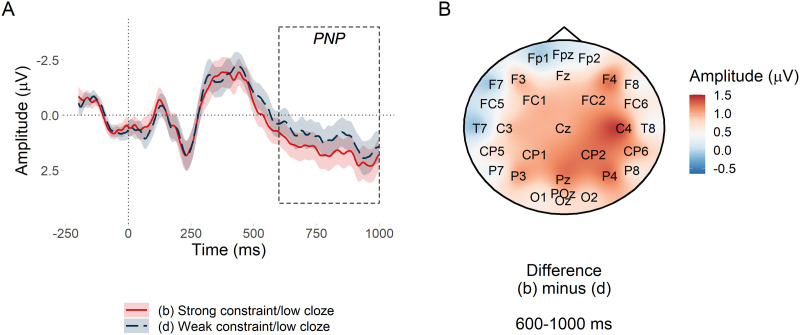
PNP constraint effect at low predictability nouns. (A) Mean amplitude at the target low probability noun in the anterior region of interest. Since constraint in the statistical analysis was represented by the continuous predictor entropy, conditions (b) and (d) are divided by the median split of their entropy values. Ribbons indicate 95% confidence intervals. (B) Subtraction plot of mean amplitude at low predictability target words between high and low median split entropy.

Figure 4A plots mean amplitude at the target word in the anterior region of interest. The PNP was most positive for low probability words in low entropy (strongly constraining) contexts and became less positive as entropy increased (constraint weakened) by a estimated mean amplitude of −0.26 *μ*V per bit of entropy, with a 95% credible interval of [−0.48, −0.05] *μ*V. Credible intervals reported throughout the manuscript are quantile-based. The Bayes factor indicated strong evidence for *H*_1_ over *H*_0_, *BF*_10_ = 17.17, consistent with Federmeier et al. (2007) and Kuperberg et al. (2020). However, those studies predicted that the effect would be centred over anterior electrodes, whereas Figure 4B suggests that in the current study, the scalp distribution of the constraint effect was centred over posterior electrodes; we return to this in the exploratory analyses. Sensitivity analyses testing the sensitivity of the Bayes factor to the choice of prior for all pre-registered analyses are presented in Appendix C in the Supporting Information.

##### N400 window.

**Figure 5.**
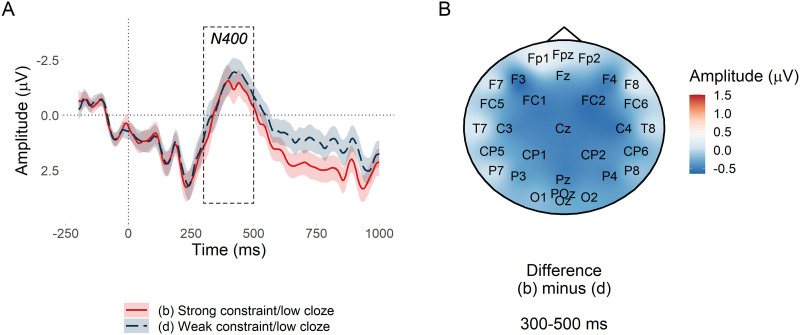
N400 constraint effect at low predictability nouns. (A) Mean amplitude at the target low probability noun in the posterior region of interest. Conditions (b) and (d) are divided by the median split of their entropy values. Ribbons indicate 95% confidence intervals. (B) Subtraction plot of mean amplitude between the high and low constraint low predictability target words. Conditions (b) and (d) are divided by the median split of their entropy values.

Our pre-registered analysis yielded inconclusive evidence about the effect of constraint in the N400 window, $\hat{\beta }$ = −0.09 [−0.30, 0.12] *μ*V, *BF*_10_ = 0.76. We attribute the inconclusive result to what appears to be between-condition differences in the behaviour of the N400 prior to and after its peak amplitude, as can be seen in Figure 5A. Prior to the peak, there was no visible effect of constraint. Past the peak however, from about 400 ms, there appeared to be a small constraint effect, which could be consistent with the beginning of post-N400 processing. Alternatively, it could reflect differences in mean latency of the N400 between the two conditions, with one condition peaking slightly later and thus having a higher amplitude for longer. (We thank a reviewer for this suggestion.) Figure 5B shows a very small difference between high and low entropy in the N400 window with a topographic distribution typical of the N400.

#### Effect of differing predictability at the noun under strong constraint

##### PNP window.

**Figure 6.**
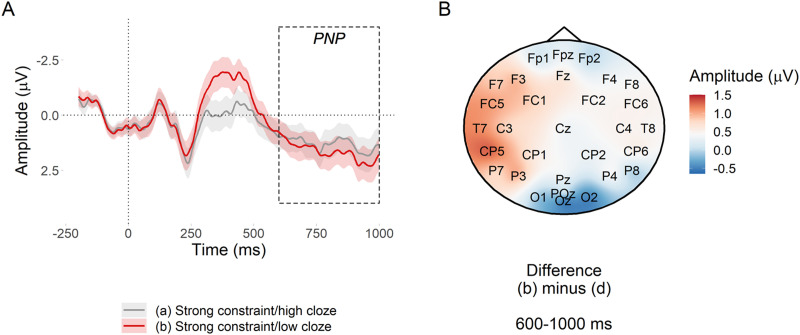
PNP predictability effect at nouns in strongly constraining contexts. (A) Mean amplitude at the target noun in the posterior region of interest. Ribbons indicate 95% confidence intervals. (B) Subtraction plot of mean amplitude between the high and low predictability target words.

Figure 6A suggests a small predictability effect in the expected direction with respect to Kuperberg et al. (2020), but the evidence was inconclusive, $\hat{\beta }$ = −0.11 [−0.24, −0.01] *μ*V, *BF*_10_ = 1.67. However, Figure 6B suggests that there may have been a more left lateralised predictability effect; a similar predictability effect was also observed in Kuperberg et al. (2020) but was not analysed separately.

##### N400 window.

**Figure 7.**
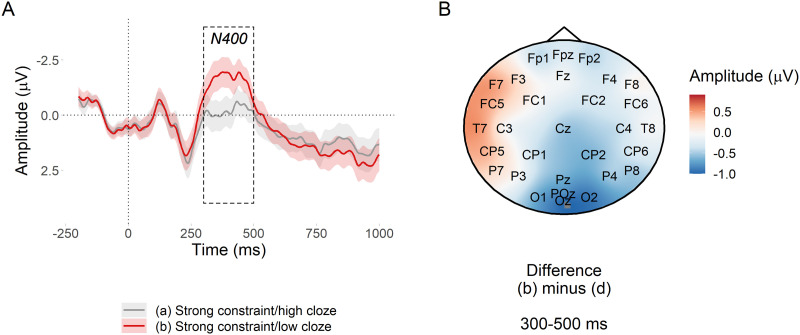
N400 predictability effect at nouns in strongly constraining contexts. (A) Mean amplitude at the target noun in the posterior region of interest. Ribbons indicate 95% confidence intervals. (B) Subtraction plot of mean amplitude between the high and low predictability target words.

Low probability words in strongly constraining contexts elicited a large N400 in comparison to high probability words (Figure 7). There was extremely strong evidence for the effect, $\hat{\beta }$ = 0.56 [0.41, 0.71] *μ*V, *BF*_10_ > 20^7^.

## DISCUSSION

Using the pre-registered analysis plan, we observed strong evidence that low probability words elicited more positive amplitude in the post-N400 window in strongly versus weakly constraining contexts. The direction of this effect was in line with previous research (Federmeier et al., 2007; Kuperberg et al., 2020), but its scalp distribution was consistent with a posterior P600 and not an anterior PNP. The effect of predictability in the PNP window was inconclusive, which contradicts Kuperberg et al. (2020). The N400 window was more consistent with previous research: Although between-condition differences in the behaviour of the N400 before and after its peak amplitude were apparent in the latter part of the window, it did not appear that constraint affected the N400 (Federmeier et al., 2007; Federmeier & Kutas, 1999; Kuperberg et al., 2020; Lai et al., 2021; Szewczyk & Schriefers, 2013; Thornhill & Van Petten, 2012) and there was strong evidence for the standard N400 predictability effect (Kutas & Federmeier, 2011).

These findings support our hypotheses only partially. In support of our hypotheses, the constraint effect was apparent in the post-N400 window and not in the N400 window. This demonstrates a dissociated effect of probabilistic representation strength as processing progresses over time: It does not appear to affect initial semantic processing in 300–500 ms window (Kutas & Federmeier, 2011; Rabovsky et al., 2018), but it does appear to affect the downstream consequences of this processing in the 600–1,000 ms window. Contrary to our hypotheses, the topography of the late positive effect was more consistent with a P600 than with the PNP reported in the literature. The P600 has been associated with conflict monitoring and syntactic reanalysis—a different type of processing than that proposed for the PNP (Bornkessel-Schlesewsky & Schlesewsky, 2008; Brouwer et al., 2017; Fitz & Chang, 2019; Kim & Osterhout, 2005; Kuperberg et al., 2003; Osterhout & Holcomb, 1992).

Since a constraint effect on the P600 was unexpected in the current design, in the following section we first establish statistical evidence for the effect. We also examine whether word predictability affected the P600, since it was shown to affect the PNP in the previous research we had been trying to replicate. We then present a number of exploratory analyses probing different factors that could have resulted in the observed constraint effect being posterior (P600) rather than anterior (PNP).

In other exploratory analyses, we examine the two effects for which we did not find conclusive evidence—the PNP predictability effect and the N400 constraint effect—and simulate data sets with larger sample sizes to determine what a sufficient sample size would have to be to yield conclusive evidence. Finally, we analyse the stop signal task to determine whether participants who were better at suppressing motor responses also showed larger constraint-based PNPs or P600s. We turn now to these exploratory analyses.

### Exploratory Analysis

#### Statistical evidence for the P600 constraint effect

**Figure 8.**
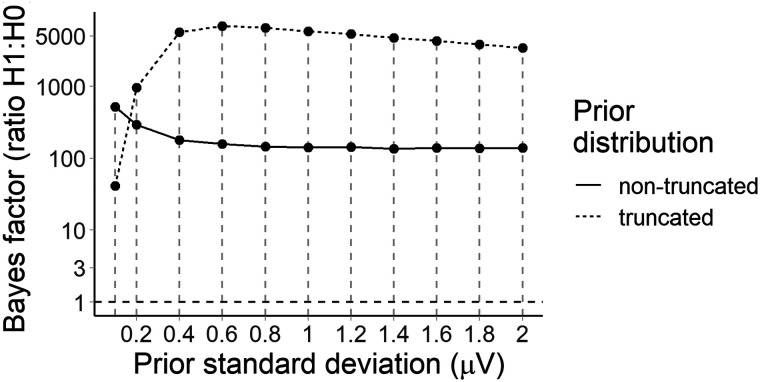
Bayes factors for the P600 constraint effect under a range of priors. The dashed line at a Bayes factor of 1 indicates equivalent evidence for *H*_1_ and *H*_0_. Bayes factors above this line indicate evidence in favour of *H*_1_, with Bayes factors of over 10 generally considered to indicate strong evidence (Jeffreys, 1939).

We analysed average amplitude in the 600–1,000 ms across the posterior region of interest (electrodes Cz, CP1, CP2, P3, Pz, P4, and POz). The model was that used for the PNP, but since we did not have a priori hypotheses about the direction or magnitude of the constraint effect, we examined a range of priors. Figure 8 suggests that there was strong evidence (*BF*_10_ from 41 to 5,472) that low probability words elicited a more positive P600 in strong versus weak constraint regardless of prior, although the Bayes factor peaked around a prior standard deviation of 0.6 *μ*V (truncated to assume a negative effect), $\hat{\beta }$ = −0.60 [−0.86, −0.34] *μ*V.

#### Predictability and the posterior P600

**Figure 9.**
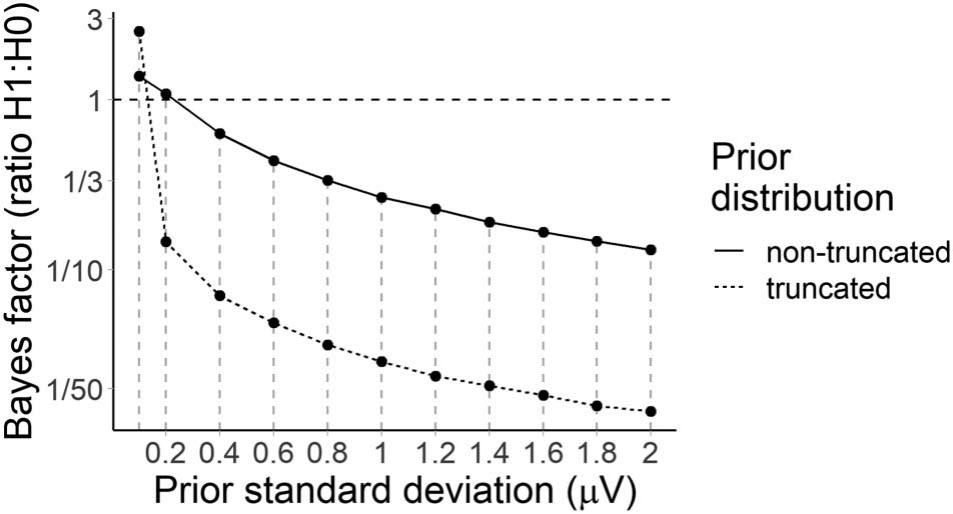
Bayes factors for the P600 predictability effect under a range of priors. The horizontal dashed line at a Bayes factor of 1 indicates equivocal evidence for *H*_1_ and *H*_0_. Above this line, evidence increases for *H*_1_, below this line, for *H*_0_. Evidence above 10 for *H*_1_ or below 1/10 for *H*_0_ is generally considered to be strong. The plot panels show the estimated ratio of evidence for *H*_1_ over *H*_0_ (*BF*_10_).

In a previous study, both contextual constraint and word predictability affected the PNP (Kuperberg et al., 2020). Assuming that a similar underlying process drove the P600 constraint effect in the current study, we additionally tested the effect of predictability in the 600–1,000 ms window. We fit the same model as used to test the PNP predictability effect, but used mean amplitude across posterior electrodes Cz, CP1, CP2, P3, Pz, P4, and POz. We used a range of priors and computed a Bayes factor for each. Figure 9 suggests that for prior standard deviations of 0.2 *μ*V or more that assumed a negative effect, there was strong evidence against a predictability effect, $\hat{\beta }$ = −0.11 [−0.24, −0.01] *μ*V, prior: *β* ∼ *Normal*_−_(0, 0.2). For priors that made no assumption about the direction of the effect, evidence against a predictability effect was weaker, but tended in the same direction as for priors assuming a positive effect.

#### How many subjects would have been needed to yield conclusive evidence?

**Figure 10.**
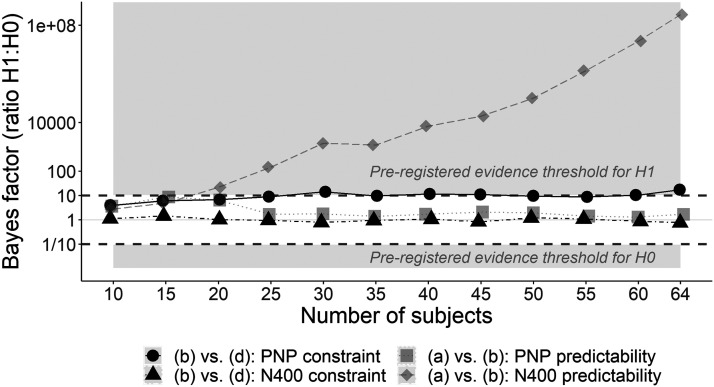
Ratio of evidence for *H*_1_:*H*_0_ (Bayes factor) as sample size increases. The key contrasts regarding the effect of constraint on the PNP and N400 are in black.

Using our pre-registered analysis plan, we were unable to find conclusive evidence for two of our four pre-registered hypotheses. Figure 10 plots the Bayes factor for each of our four comparisons as sample size increased. Our two key comparisons are highlighted in black. Despite the Bayes factor remaining inconclusive for one of these key comparisons—the N400 constraint effect—we ceased recruitment due to the difficulty in recruiting participants during the COVID-19 pandemic. The post-peak N400 constraint-related differences 614 may also have prevented the Bayes factor from ever being able to distinguish between null and alternative hypotheses, even if we had reached our pre-registered cap of 150 participants, which would have been infeasible given the poor recruitment rate.

**Figure 11.**
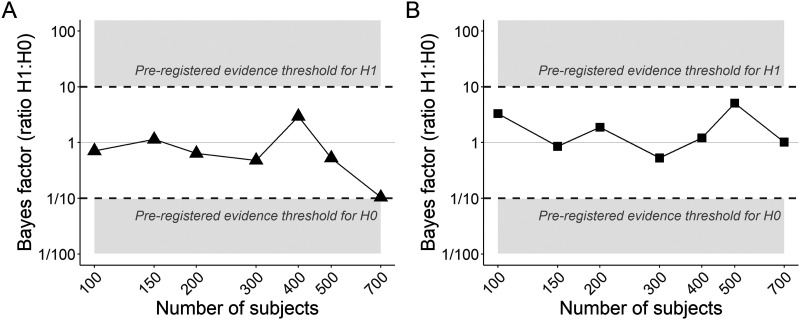
Bayes factors at simulated sample sizes. (A) N400 constraint effect: One data set was simulated for each sample size to which the pre-registered analysis model was fit. Each point in the plot reflects the Bayes factor for that sample size. (B) PNP predictability effect: Each point reflects the Bayes factor for a pre-registered analysis applied to a simulated data set.

We therefore conducted a design analysis (Gelman & Carlin, 2014) to determine how many participants would be needed in a future experiment to yield conclusive evidence for the null hypothesis. We assumed that the estimates from the final sample of 64 participants reflected true values and used them to simulate new data sets for between 100 and 700 participants. A Bayes factor for the N400 constraint effect was computed for each sample size. Figure 11A suggests that even with the pre-registered cap of 150 participants, we would not have furnished strong evidence against the constraint effect on the N400 using our pre-registered analysis plan. The analysis suggested that, assuming that the estimates obtained from the present data are indeed the true values, at least 700 participants would be needed to demonstrate strong evidence against a constraint effect using the current experimental design.

Since our secondary hypothesis about the PNP predictability effect also yielded inconclusive evidence with 64 participants, we repeated the same design analysis and noted that again, assuming our parameter estimates reflected the ground truth, the pre-registered cap of 150 participants would not have yielded conclusive evidence using the current design. Figure 13B suggests that if there were a true predictability effect, not even 700 participants would have been sufficient to yield conclusive evidence for it.

#### Factors that could have changed the scalp appearance of the constraint effect or its underlying cognitive process

##### Individual variability.

**Figure 12.**
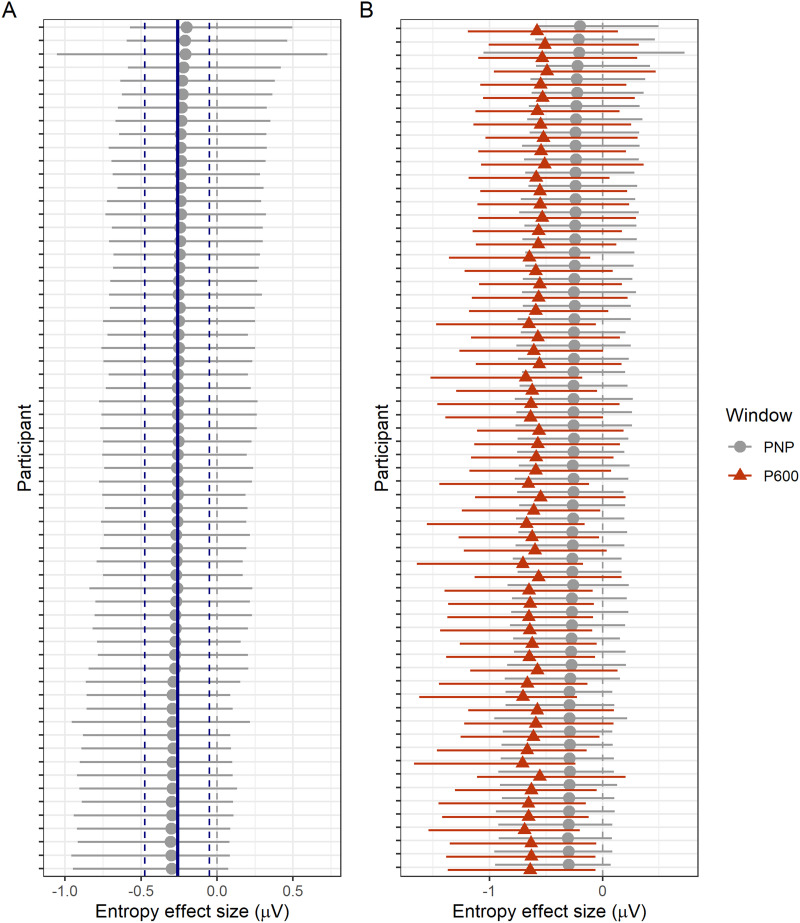
Individual posterior estimates of the effect of entropy in the post-N400 window. (A) Individual posteriors from the pre-registered model of the anterior PNP (grey) are plotted against the group estimate (blue). Points show posterior means and error bars the 95% credible intervals. (B) Individual posterior estimates for the PNP (grey) are overlaid with estimates from the model fit to P600 amplitudes at the top of this section (orange).

The scalp topography of an averaged ERP can be affected by factors such as variability in cortical folding and skull thickness between participants (Luck, 2005a). We examined individual variability by plotting posterior estimates of the entropy effect by participant for the PNP (Figure 12A) and P600 (Figure 12B). However, individual estimates largely reflected the group mean with no obvious systematic outliers.

Another possibility is that individual participants differed in their response to the unexpected word: Some may have suppressed the disconfirmed sentence parse (PNP), while others attempted to reanalyse the sentence (P600). If this were the case and we simply had more P600-type processors in our participant pool, one could expect a crossover effect where participants with smaller PNP constraint effects showed larger P600 constraint effects, and vice versa. Individual PNP estimates are plotted against P600 estimates in Figure 12B, but do not suggest a crossover effect. To quantify the relationship between individual PNP and P600 constraint effects, we fit a multivariate linear mixed effects model with the same form as the constraint models above, except that there were two response variables: mean amplitude in the PNP and in the P600 windows/regions. A prior for the correlation of the PNP and P600 constraint effects was also added: *LKJ*(2). A crossover between the PNP and P600 constraint effects would yield a negative correlation estimate; instead, the model suggested a positive correlation, $\hat{\rho }$ = 0.61 [0.60, 0.63]. In other words, participants with larger PNPs also tended to exhibit larger P600s.

##### The operationalisation of constraint as entropy.

**Figure 13.**
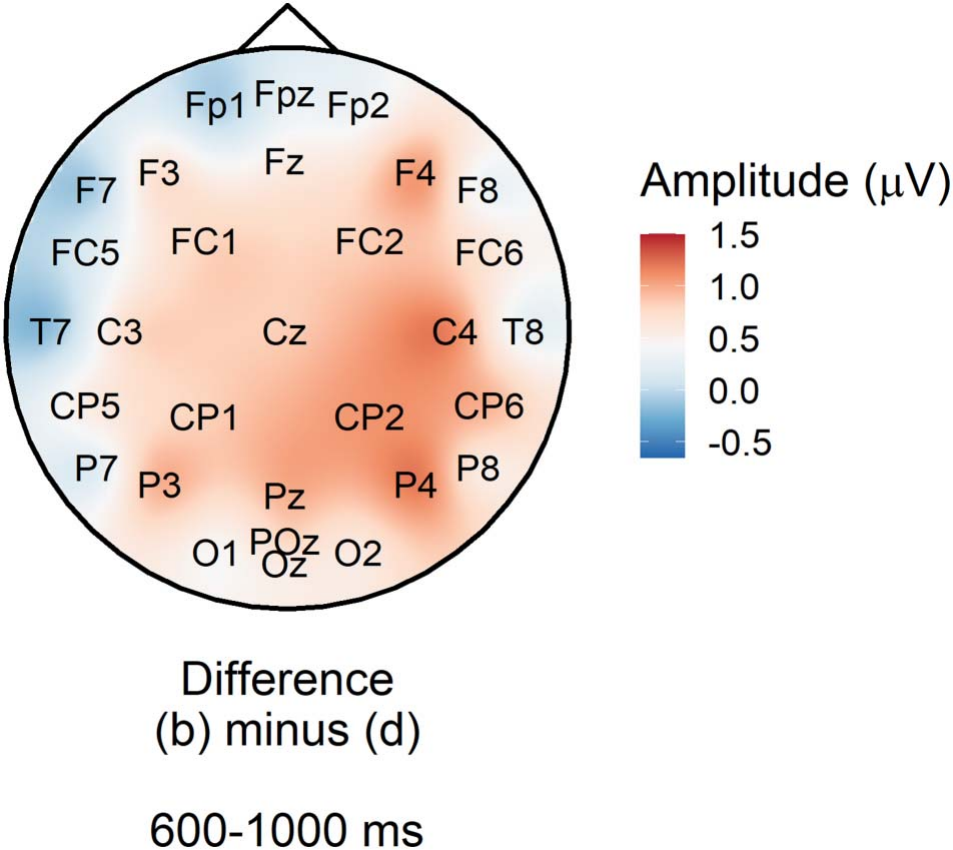
Subtraction plot of mean amplitude at low predictability target words between high and low constraint as defined by category rather than entropy.

A major difference between the current study and Kuperberg et al. (2020) and Federmeier et al. (2007) is the use of entropy as a continuous measure of constraint. Instead, as in those studies, we could have used cloze probability of the most often given response, which, in the high constraint condition (b) was 0.80, 95% range = [0.50, 1.00] and in the low constraint condition (d), 0.10, 95% range = [0.06, 0.50]. To determine whether a categorical definition of constraint would have changed the topography of the constraint effect, we re-plotted Figure 4B by subtracting condition (b) from condition (d) as defined by their category, rather than by a median split of entropy values. As can be seen in Figure 13, the distribution of the constraint effect was still posteriorly focused and was actually lower in magnitude.

##### Semantic association of target nouns with their context.

Another difference between the current study and Kuperberg et al. (2020) is that there was a semantic association between the target noun and its preceding context. Kuperberg et al. (2020) deliberately kept semantic association low. Assuming that low semantic association means weaker preactivation of the target word by the context, it could be that readers in Kuperberg et al. had to work harder to update their sentence representation at the unexpected noun than participants in the current study, and that this extra work was necessary to elicit a detectable PNP constraint effect. If so, we could expect that low semantic association is a necessary condition for eliciting the constraint effect. In Table 2, we compare semantic association of target nouns and their contexts across three studies: the current study, Kuperberg et al. (2020), and Federmeier et al. (2007). For our own stimuli, we computed cosine similarity using the LSAfun package in R (Version 0.6.3; Günther et al., 2015). We used a pretrained German latent semantic analysis (LSA) space with 300 dimensions (Günther, 2022) created from the 1.7 billion-word deWaC corpus (Baroni et al., 2009). Kuperberg et al. (2020) also computed cosine similarities using LSA, and we present the values reported in their paper. For Federmeier et al. (2007), we computed cosine similarities using LSAfun and a pretrained English LSA space with 300 dimensions (Günther, 2022) created using the British National Corpus, the ukWaC corpus (Baroni et al., 2009), and a 2009 Wikipedia dump. (We thank Kara Federmeier for providing the stimuli.)

**Table 2.** Cosine similarity of target nouns with their context.

Condition	Current study		Kuperberg et al. (2020)		Federmeier et al. (2007)	
	Mean	95% range	Mean	95% CI	Mean	95% range
a) Strong constraint, high cloze	0.40	0.17, 0.61	0.18	0.10, 0.26	0.40	0.18, 0.64
b) Strong constraint, low cloze	0.36	0.17, 0.58	0.01	−0.01, 0.03	0.33	0.17, 0.52
c) Weak constraint, low cloze	0.34	0.13, 0.54	–	–	0.36	0.14, 0.59
d) Weak constraint, low cloze	0.33	0.15, 0.56	0.01	−0.01, 0.03	0.34	0.12, 0.56

*Note*. Condition names for all studies are presented in line with condition names from the current study.

While semantic association in the current study was notably higher than in Kuperberg et al. (2020), it was comparable with Federmeier et al. (2007), and yet Federmeier et al. saw a distinct PNP constraint effect and no associated P600 effect. The degree of semantic association between target noun and context thus may not explain our findings.

In the current experiment, it was possible to quantify whether cosine similarity affected whether a constraint-based anterior PNP or posterior P600 effect was seen using our model of the potential crossover effect above. We fit the same multivariate linear mixed effects model with the two response variables mean amplitude in the PNP and P600 windows/regions, but added scaled and centred cosine similarity as a predictor interacting with entropy. The main effect of cosine similarity was not consistent with a change in amplitude, $\hat{\beta }$*_PNP_* = 0.10 [−0.11, 0.32] *μ*V, $\hat{\beta }$_*P*600_ = −0.12 [−0.35, 0.11] *μ*V, nor was its interaction with entropy, $\hat{\beta }$*_PNP_* = 0.02 [−0.21, 0.24] *μ*V, $\hat{\beta }$_*P*600_ = −0.05 [−0.26, 0.17] *μ*V. As before, the model yielded a strong positive correlation between the PNP and the P600, $\hat{\rho }$ = 0.61 [0.59, 0.63], suggesting that readers who exhibited larger PNPs still exhibited larger P600s, even after semantic relatedness was taken into consideration.

##### Task-related effects.

**Figure 14.**
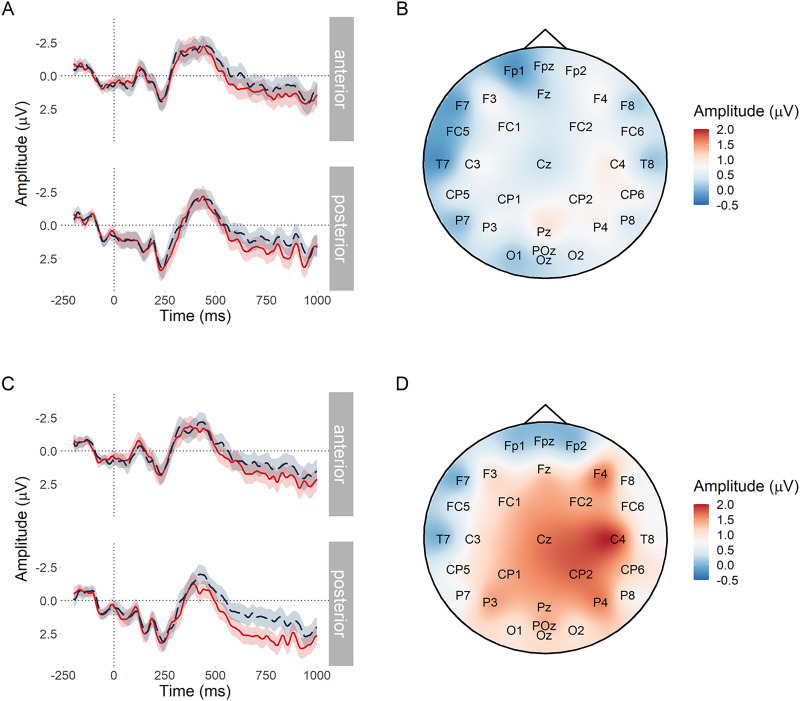
Comparison of post-N400 amplitude at target nouns on trials after a trial where a question was asked versus where no question was asked. (A) ERP amplitude in the anterior and posterior scalp regions on trials following a question. (B) Topography of the constraint comparison on trials following a question (strong minus weak constraint via median split entropy). (C) ERP amplitude in the anterior and posterior scalp regions on trials following a no-question trial. (D) Topography of the constraint comparison on trials following a no-question trial (strong minus weak constraint via median split entropy).

One of the factors that may play a role in the topography of positive components in the post-N400 window is the type of task (Friederici et al., 2002; Kuperberg & Brothers, 2019). During our experiment, participants answered a yes/no question after 50% of sentences (28 sentences per condition). In Figure 14, we compare topography and mean ERP amplitude in the late window between target nouns that appeared in a sentence directly following a sentence that was one of the 50% of question trials (Figure 14A and B), with nouns that appeared after a sentence with no question (Figure 14C and D). Conditions (b) and (d) have been collapsed and split into high and low constraint by their median entropy value. The posterior P600 effect is markedly smaller in trials following a question (Figure 14B vs. Figure 14D), suggesting readers behaved differently when they may have expected another question versus when they did not.

Participants’ expectations with respect to an upcoming question could have had various effects on their processing. For example, although questions were randomly distributed, participants may have thought that question trials were more likely to appear immediately after no-question trials and focussed more on the sentences, enhancing their conflict-detection response and eliciting the P600 constraint effect after no-question trials (Figure 14D). Alternatively, participants may have been primed to expect another question trial if they had just seen one, and engaged a more PNP-type of processing such as suppressing information not relevant to answering the question. This could explain the absence of the P600 in post-question trials, although there was no suggestion of a PNP in Figure 14B. Using the same model and priors as for the pre-registered PNP constraint analysis, there was only inconclusive statistical evidence for the anterior PNP constraint effect in the post-question trials, $\hat{\beta }$ = −0.23 [−0.49, −0.02] *μ*V, *BF*_10_ = 4. When compared with the strong evidence for the same effect when all trials were used (see main pre-registered analysis), this finding does not suggest a functional dissociation between the PNP and P600 on post-question and post-no-question trials.

##### Trial order effects.

**Figure 15.**
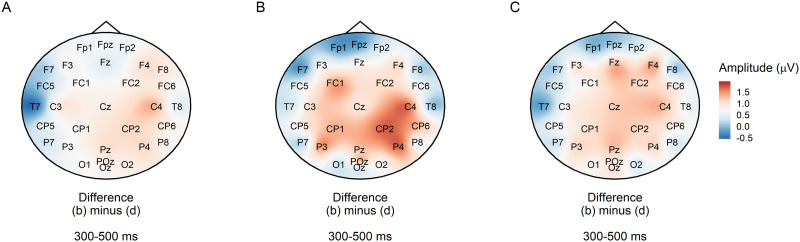
Comparison of post-N400 amplitude at target nouns in different stages of the experiment. (A) First third of the experiment. (B) Middle third of the experiment. (C) Final third of the experiment.

The absence of an anterior PNP may have been due to participants not having engaged in predictive processing once they got used to or guessed the purpose of the experiment. If so, this may have been visible across the experiment, e.g., with an anterior PNP early on when participants were still predicting, and a posterior P600 later as prediction stopped. Figure 15 suggests this was not the case, with no PNP apparent at any stage of the experiment. We quantified a trial order effect by adding trial number as an interaction with entropy to our pre-registered constraint model. We fit two separate models, one of amplitude in the anterior region of interest (PNP) and one of amplitude in the posterior region (P600). There appeared to be a main effect of trial order in the anterior region, with amplitude becoming less positive as the experiment progressed, $\hat{\beta }$ = −0.14 [−0.36, 0.07], but this did not interact with entropy, $\hat{\beta }$ = 0.005 [−0.25, 0.27]. In other words, there was no suggestion that a constraint effect on the PNP differed across the experiment. In the posterior region, there appeared to be neither a main effect of trial order, $\hat{\beta }$ = −0.04 [−0.26, 0.16], nor an interaction of trial order with entropy, $\hat{\beta }$ = 0.11 [−0.15, 0.37].

##### The choice of temporal filter.

One ERP preprocessing step that can potentially alter the appearance of ERP components is the choice of filter (Luck, 2005a; Tanner et al., 2015; Vanrullen, 2011). Filter choice can create artificial differences, usually in the temporal appearance of ERP components, where amplitude from one time window is “smeared” into another as an artifact of the filtering process. The degree of smear depends on various filter settings and filter types, and can affect things like component overlap, which may have been present in our N400 window. Although smearing is more likely to affect the magnitude of an effect rather than its topography, we compared two different filter types. For our pre-registered preprocessing pipeline, we used FIR filters, but another common choice is infinite impulse response (IIR) filters. We re-preprocessed the data using a Butterworth zero-phase (two-pass forward and reverse) non-causal IIR filter with filter order 16 (effective, after forward–backward) and cut-offs at 0.01 and 30 Hz (−6.02 dB).

**Figure 16.**
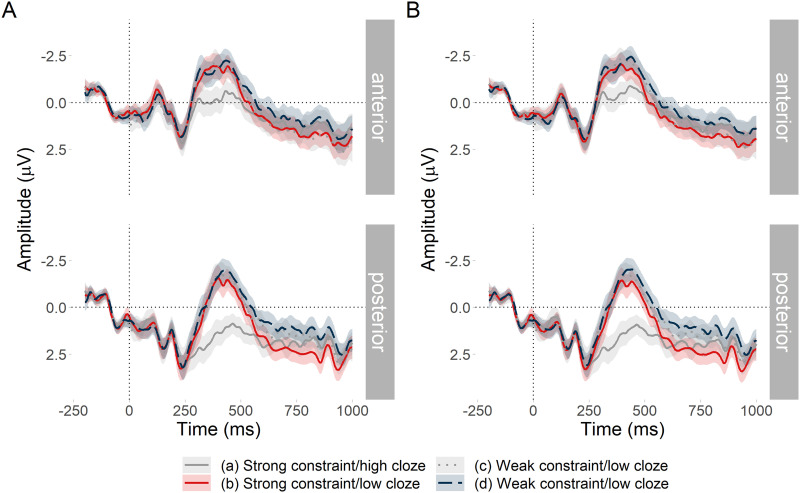
Comparison of finite impulse response (FIR) and infinite impulse response (IIR) filters on the entropy effect in the post-N400 window. (A) Mean amplitude over time at target words after preprocessing using FIR filters. (B) Mean amplitude over time after preprocessing using IIR filters.

ERPs after both types of preprocessing are plotted in Figure 16. Figure 16A shows the ERP using FIR filters (Figure 4B in the main text) and Figure 16B the ERP using IIR filters. We observed small differences in the amplitude of the ERP signal in each of our analysis windows, but nothing of a degree that would have changed our conclusions.

#### Correlation of post-N400 amplitude with the stop signal task

In a final exploratory analysis, we examined whether performance on a response inhibition task would predict the magnitude of the PNP constraint effect, with the hypothesis that better inhibitors might elicit larger PNPs. Before undergoing the EEG recording, participants completed a stop signal task. Participants saw either a circle or a square on a screen 765 and were instructed to press the “J” key on a keyboard as soon as they saw a circle and the “F” key as soon as they saw a square, unless they heard a tone presented via headphones, in which case they should not press anything. Our exploratory hypothesis was that participants who performed better at suppressing their responses after stop signals might also show larger PNP effects, if in fact the PNP were related to suppression. The stop signal tone was a 750 Hz sine wave tone presented for 75 ms with no attack or decay. The stop signal varied in its delay after the visual presentation, determined via a tracking procedure: The starting delay was 250 ms and 50 ms was subtracted after unsuccessful stop trials (i.e., trials where a response was made despite hearing the tone), and 50 ms added after successful stop trials. The minimum stop signal delay was 50 ms and the maximum, 1,000 ms. The mean stop signal delay was 525 ms, 95% CI = [511, 539] ms (see Table 3 for further descriptive statistics).

**Table 3.** Stop signal task descriptive statistics.

Measure	Mean	95% CI
Probability of no response on go trial	0.06	0.04, 0.08
Probability of response on stop trial	0.20	0.18, 0.21
Mean SSD	527	514, 541
SSRT	245	230, 260
RT on go trials	896	872, 920
RT on stop trials	781	754, 809

*Note*. Means and 95% confidence intervals are presented for the probability of (incorrectly) not responding on a go trial, the probability of (incorrectly) making any response on a stop trial, stop signal delay after visual presentation (SSD), stop signal reaction time (SSRT), reaction time (RT) of any response on go trials, and reaction time (RT) of any response on stop trials.

Participants were given four practice trials. The main experiment contained eight trials per block and three blocks. Each block contained four circles and four squares presented in random order. Stop signals were presented after one of the squares and one of the circles. Each trial began with a fixation dot presented for 250 ms, followed by the visual presentation. A keyboard response to the visual presentation triggered a blank screen of 500 ms duration and the beginning of the next trial. If no response was made, the next trial began after a timeout of 1,250 ms. At the end of each block, participants were given feedback about their proportions of incorrect responses, missed responses, and correctly suppressed responses, as well as their average reaction time. The duration of the feedback screen was determined by participants. The task was presented using OpenSesame (Mathôt et al., 2012) on a 56 cm monitor in a sound-insulated cabin.

Of the 64 participants whose EEG was recorded, 59 had useable stop signal data: One participant was excluded as they were unable to understand the stop signal task, and two participants’ stop signal data were not saved in error. Two further participants were excluded because their mean response time on go trials was more than two standard deviations faster or slower than stop trials, violating the assumptions of the stop signal reaction time calculation (Verbruggen et al., 2019). Stop signal reaction time (SSRT) was calculated via the integration method in Verbruggen et al. (2019).

We used SSRT as a predictor of amplitude in two separate models, one for the PNP and one for the P600. We used the same model specification as for the main analysis, but added log transformed SSRT as a continuous predictor interacting with entropy. All predictors were scaled and centred. Since there was only one SSRT observation per participant, random slopes were not estimated. With respect to the prior, we had no a priori expectation about the direction in which SSRT would affect amplitude: Faster SSRTs (better response inhibition) could hypothetically result in either a more marked inhibitory response to unexpected input and higher amplitude, or a more efficient inhibitory response and lower amplitude. We also did not expect that the effect of SSRT would be any larger than that of entropy or cloze probability. We therefore used the same prior for SSRT as for entropy and cloze probability, only nontruncated: *Normal*(0, 0.2) *μ*V. Due to the mix of truncated and nontruncated priors on the predictors, which brms did not allow at the time of analysis, the model was fit in the RStan R package (Stan Development Team, 2018, 2020).

The posterior estimates of the interaction of entropy and SSRT on both PNP and P600 amplitude were both centred around zero, $\hat{\beta }$*_PNP_* = 0.09 [−0.12, 0.32] *μ*V and $\hat{\beta }$_*P*600_ = 0.05 [−0.18, 0.28] *μ*V, which was not consistent with faster SSRTs being predictive of amplitude, regardless of constraint. Estimates were also not consistent with a main effect of SSRT on amplitude in either the anterior, $\hat{\beta }$*_PNP_* = 0.11 [−0.20, 0.41] *μ*V, or the posterior scalp region, $\hat{\beta }$_*P*600_ = −0.03 [−0.30, 0.25] *μ*V. In sum, the data were not suggestive that faster performance on the stop signal task was associated with either PNP or P600 amplitude. However, accuracy on the stop signal task was too high according to guidelines set out by Verbruggen et al. (2019), which violates some assumptions in computing SSRT. More specifically, the probability of responding after a stop signal should be around 0.50, or at least between 0.25 and 0.75; our participants had a mean probability of 0.20. The finding should thus be taken with caution.

## GENERAL DISCUSSION

Our study addressed the idea that encountering a low predictability noun in a context where a different noun was highly predictable should trigger greater processing cost than a low probability noun in a context where no particular noun was predictable. We set out to conceptually replicate the finding that a contextual constraint-based processing cost at unexpected but still plausible words is reflected by an increase in anterior PNP amplitude (Federmeier et al., 2007; Kuperberg et al., 2020). Using an experimental design that maximised our ability to detect constraint effects and a sample size determined by reaching a threshold for strong evidence, we were able to partially replicate previous findings. We observed strong evidence that low probability words elicited more positive amplitude in the post-N400 window in strongly versus weakly constraining contexts, but the scalp distribution of this positivity was consistent with a posterior P600 and not an anterior PNP. Also in contrast with previous findings (Kuperberg et al., 2020), the effect of predictability in the post-N400 window was inconclusive, both for the PNP and the P600. This suggests that the critical factor in determining processing at the target noun was not how predictable that specific noun was, but rather how strongly the preceding context had driven expectations about the event as a whole in which the target noun, and also other words or concepts, might be expected. Findings in the N400 window were highly consistent with previous research: Constraint did not appear to affect the N400 (Federmeier et al., 2007; Federmeier & Kutas, 1999; Kuperberg et al., 2020; Lai et al., 2021; Szewczyk & Schriefers, 2013; Thornhill & Van Petten, 2012), and there was strong evidence for the standard N400 predictability effect (Kutas & Federmeier, 2011).

### Is the PNP Affected by Contextual Constraint?

The anterior PNP is proposed to be a distinct ERP phenomenon reflecting the cost of shifting the interpretation of a sentence after unexpected input, becoming larger when the preceding context increases certainty about a particular interpretation (Federmeier et al., 2007; Kuperberg et al., 2020). We note here an assumption: that increased ERP amplitude in one condition relative to another can be interpreted as increased processing cost in the higher amplitude condition. However, a cost-amplitude association may not reflect the true state of affairs since latency variability can create the appearance of artificial amplitude differences (Luck, 2005b). The precise link between ERPs and neuronal activity is also still unclear. However, for the purposes of this paper, we assume a cost-amplitude link, based on the typical pattern that more “difficult” tasks (e.g., dealing with semantically unexpected words or odd syntax) reliably increase the amplitude of at least the N400 and late positive components.

The mechanism underlying the PNP is proposed to be separate from that of another post-N400 positive component—the posterior P600—since in two previous studies only the PNP was affected by a constraint manipulation at plausible but unexpected words and not the P600 (Federmeier et al., 2007; Kuperberg et al., 2020). In one of these studies, the reverse observation was made for words that were anomalous in their contexts: Constraint affected the P600 but not the PNP (Kuperberg et al., 2020). Together, these findings have been taken to suggest that the PNP reflects the successful update of a sentence representation with plausible input and the P600 an error signal triggered by implausible input. The current findings contrast with Kuperberg et al. and Federmeier et al. in two ways: First, we did not observe a constraint effect for plausible words in the anterior PNP but rather in the posterior P600, and second, the effect on the P600 was elicited by plausible unexpected words. In this section we examine a number of possible explanations for the contrasting findings.

With respect to the posterior rather than the anterior distribution of the constraint effect, we ruled out with exploratory analyses that the difference was related to our definition of constraint, or to individual variability in constraint effects. Since the type of filter used during EEG preprocessing can also alter at least the temporal appearance of ERPs (Luck, 2005a; Tanner et al., 2015; Vanrullen, 2011), we additionally re-preprocessed the data using a different filter, but the topography of the constraint effect remained posterior. The combination of filter settings and the choice of baseline can create artificial differences in ERP topography (Tanner et al., 2016): We used average amplitude over a pre-stimulus period of 200 ms as a baseline and a bandpass filter of 0.01–30 Hz. Of the previous studies in which constraint was examined, all used 100 or 200 ms pre-stimulus baselines (100 ms for all but two studies), with which effects on the PNP both were and were not observed; that is, there was no systematic effect of the baseline duration on whether or not a PNP constraint effect was observed. Almost every study used different bandpass filter settings, which—while of concern for ERP research more broadly—again does not suggest a systematic effect on the appearance of the PNP (although we did not manipulate these settings directly and so cannot rule it out).

The type of task that participants do during the EEG recording can also affect the appearance, including the topography, of late positive components (Friederici et al., 2002; Kuperberg & Brothers, 2019; Sassenhagen & Bornkessel-Schlesewsky, 2015; Sassenhagen et al., 2014), so we reviewed task types among previous studies. Participants in the current study answer yes/no comprehension questions after 50% of sentences. In previous studies where a constraint effect on the anterior PNP was observed (but not on the posterior P600), participants had to judge whether each sentence “made sense” and additionally answer yes/no questions about filler sentences (Kuperberg et al., 2020), or had no task during the experiment but were informed they would complete a word recognition task after the experiment (Federmeier et al., 2007). Of the previous studies that have observed no or contrasting effects of constraint on the PNP/P600, participants either had to indicate after each sentence whether a probe word appeared in that sentence (Thornhill & Van Petten, 2012), or they were informed they would complete a word recognition task after the experiment (Federmeier & Kutas, 1999; Lai et al., 2021; Szewczyk & Schriefers, 2013; Wlotko & Federmeier, 2007). Thus, there did not appear to be systematic differences in task type between studies. In addition, we did not observe statistical evidence that the presence or absence of a question influenced whether participants exhibited a PNP or P600 in the current study. Future studies directly manipulating the effect of task type on eliciting the PNP versus the P600 would better address this question, however.

With respect to the P600 being elicited by plausible words, this is somewhat unusual since the target noun and context were also syntactically well formed and the P600 has traditionally been associated with reanalysis after syntactic violations (Hagoort et al., 1993; Osterhout & Holcomb, 1992). However, P600s are also reliably observed at the verb in role-reversal sentences which are syntactically well-formed, just semantically odd, for example, *the dog that the man bit* (Kim & Osterhout, 2005; Kuperberg et al., 2003; Kuperberg et al., 2007). Van Petten and Luka (2012) also note a number of predictability studies where a P600 was elicited by plausible unexpected words that did not involve a role reversal. Thus rather than being limited to reanalysis after syntactic violations, the P600 has been proposed to signal a more general conflict detection or integration process recruiting the left inferior frontal gyrus (Brouwer et al., 2017; Brouwer & Hoeks, 2013; Fitz & Chang, 2019; van de Meerendonk et al., 2011). In our case, it likely reflects the conflict between readers’ strong event representation and the low probability input (Kuperberg et al., 2020; Laszlo & Federmeier, 2009; Vissers et al., 2006).

The combination of strong constraint and high semantic relationship between target words and their contexts in the current study are known to increase the likelihood of the P600’s appearance in syntactically well-formed sentences (Kuperberg & Brothers, 2019). Since semantic association was higher in our study than in Kuperberg et al. (2020), we reasoned that this could have contributed to the difference in topography. For example, high semantic association would mean that lexical preactivation of the presented target word by the context is stronger than when semantic association is weak, even in the low predictability conditions. Stronger semantic association and stronger preactivation in our study may not have required the engagement of PNP-related resources when a low probability word triggered a shift in interpretation. In contrast, weaker semantic association and weaker preactivation in Kuperberg et al. (2020) may have made the shift costlier and the PNP more pronounced. However, we compared semantic association between target words and their contexts in the current study against Kuperberg et al. (2020) and Federmeier et al. (2007; Table 2) and noted that semantic relationship in Federmeier et al.’s stimuli was comparable with our study—yet they observed a PNP and not a P600. Future experiments comparing plausible, low probability words with strong and weak semantic association with their contexts may yield further insights.

One likely factor contributing to the difference between the current and previous studies is that of statistical power: Fewer participants and/or fewer critical trials in previous studies may have led to a lower signal-to-noise ratio in the EEG recordings. It is a known issue in ERP research that if the signal-to-noise ratio is not sufficiently high, scalp topography can be misleading and statistical false positives can occur (Luck, 2005a; Luck & Gaspelin, 2016). False positives occur when low power leads to an overestimate of the effect size or a type M (magnitude) error (Gelman & Carlin, 2014). Type S (sign) errors may also result, explaining why at least one previous study reports a PNP constraint effect in the opposite direction (Federmeier & Kutas, 1999).

The current study therefore raises the possibility that the PNP constraint effect observed in previous studies may actually be part of a broad P600 response where lower sample size has contributed to Type M and S errors in the anterior region of the scalp. This would account for the anterior PNP constraint effect’s inconsistent appearance in previous studies despite similar experimental designs. If true, then our findings also suggest that the processing cost of strong probabilistic representations does not always result from having to update an interpretation or suppress disconfirmed interpretations after receiving conflicting input, but can instead be the cost of detecting the conflict itself.

### Why Was a Constraint-Based P600 Effect Not Observed in Previous Studies?

If the anterior PNP constraint effect really is just the edge of a P600 constraint effect, then one would expect to see a P600 constraint effect in at least some previous studies. One previous study did in fact observe a P600 constraint effect, but only at anomalous (implausible) words (Kuperberg et al., 2020). For anomalous words, the P600 became more positive for anomalous words in highly constraining contexts. This is consistent with the P600 constraint effect elicited by syntactic violations (Gunter et al., 2000; Hoeks et al., 2004); although in Hoeks et al. the effect was in the opposite direction and statistical evidence was not strong. One possibility therefore is that the anomalous sentences in Kuperberg et al. encouraged participants to treat unexpected-but-still-plausible words differently to the “real” conflict posed by anomalous words (as Kuperberg et al. hypothesised it would). In the absence of anomalous words in the current study, participants may have responded to low probability words in the same way as if they were errors. However, this would not account for why a P600 constraint effect was not observed in Federmeier et al. (2007)—who also did not have an anomalous condition—nor in other previous studies without anomalous conditions who observed contrasting or no PNP effects. This may again be due to a power issue, but we have also made suggestions above as to future research that could help to disentangle the PNP and P600.

Aside from the absence of anomalous words, another possibility is that features of the current study design encouraged conflict monitoring rather than prediction in participants. Generating predictions while reading is thought to be one of the necessary conditions for eliciting the PNP (Federmeier, 2022). It is possible that the large number of sentences and simple manipulation in the current design meant participants stopped predicting once they got used to (or even guessed the purpose of) the experiment. If this were the case, one might expect a constraint-based PNP early in the experiment and a constraint-based P600 later; we examined trial order effects and while the P600 constraint effect was visually most pronounced in the middle of the experiment, no PNP constraint effect was obvious either visually or statistically. Moreover, in order for readers to have shown a larger P600 in the strong constraint condition at the low predictability target, with all else being equal, the strong constraint of the context must have been used to generate some degree of expectation for a different upcoming word relative to the weak constraint context. This would suggest that readers were indeed predicting upcoming words. One hypothesis for a future experiment is that there is a difference between passive expectations when participants settle into a long experiment, and active predictions in more challenging experimental designs. One could imagine that the former encourages conflict-monitoring and thus a P600 response and the latter, suppression of previous predictions and a PNP response. There is some evidence that conscious prediction strategies modulate the PNP (Brothers et al., 2017), though not to the point of eliciting a P600 instead.

### The Effect of Probabilistic Strength on Processing Cost

Topography aside, the firm conclusion from the current and previous studies is that the effect of probabilistic representation strength on processing cost only becomes observable in the time window after the N400. The lack of a constraint effect in the N400 window is consistent with existing accounts of the N400 suggesting that the underlying cognitive processes are seated in the medial temporal gyral and posterior temporal areas of the ventral stream, at a time where phonetic and orthographic activation gives way to lexical retrieval and semantic unification (Friederici, 2012; Hagoort, 2013; Hickok & Poeppel, 2007; Lau et al., 2008). Retrieval and unification generate a probabilistic representation of the sentence, which in turn influences the activation of related words and concepts. Under these accounts, the N400 is only sensitive to the level of activation in this area, such that two words with the same activation level will elicit the same amplitude N400, regardless of how they came to be activated (Fitz & Chang, 2019; Hagoort et al., 2009; Kutas & Federmeier, 2011; Lau et al., 2008; Rabovsky et al., 2018). In our study, the low probability target in a strongly constraining context would have had low activation because the context suggested it was not a likely next word, whereas the low probability target in a weakly constraining context would have had low activation because the context did not suggest any particular next word. Their respective N400s were therefore of a similar amplitude.

Further down the ventral stream, in the post-N400 processing time window, is where we observed the consequences of a strong probabilistic representation. In the current study, a strong representation increased sensitivity to input that conflicted with expectations (assuming a conflict-based function of the P600). Interestingly, low predictability lexical items seen in strongly constraining contexts did not elicit conclusive differences in P600 amplitude relative to high predictability lexical items, suggesting that conflict was driven by the semantic richness of the preceding context rather than a simple unexpectedness detection. This indicates a change in processing with respect to the previous N400 window, where word predictability was important.

Source localisation of processing associated with the P600 has proven difficult (Friederici, 2011), however the P600 has been associated with a left inferior prefrontal-temporal cortical circuit (Brouwer et al., 2017; Brouwer & Hoeks, 2013), which also includes areas of the frontal inferior gyrus thought to mediate suppression of previous interpretations and possibly hints at the involvement of executive control (Hagoort, 2013; Kutas, 1993). Thus while we interpret our P600 constraint effect as a conflict signal, we do not believe our findings rule out that a shift in interpretation or suppression of previous representations occurs: We simply did not observe evidence that such a process is inevitably engaged by manipulating contextual strength, or that it is mappable to a single ERP phenomenon (for a discussion of the difficult “mapping problem” in behavioural neuroscience see Rösler, 2012). Indeed, if both processes involve the same cortical circuit at the same time, they may be difficult to disentangle without experimental methodologies better suited to spatial mapping such as magnetoencephalography and functional magnetic imaging.

### Predictability and the PNP

In contrast with constraint, word predictability is a more reliable factor in eliciting the PNP, with low probability words triggering more positive amplitudes than high probability words (Brothers et al., 2017; Brothers et al., 2020; DeLong et al., 2011; DeLong et al., 2014; Federmeier et al., 2007; Hodapp & Rabovsky, 2021; Kuperberg et al., 2020; Ness & Meltzer-Asscher, 2018; Thornhill & Van Petten, 2012). It was therefore surprising that the current study did not find stronger evidence of a predictability effect in the anterior scalp region. However, we did see a left-lateralised effect (Figure 6). Among previous studies reporting an anterior PNP predictability effect, several observed this to be distributed across frontal and/or left lateral electrodes (DeLong et al., 2011; DeLong et al., 2014; Federmeier et al., 2007; Hodapp & Rabovsky, 2021; Kuperberg et al., 2020; Szewczyk & Schriefers, 2013). One possibility is that the left lateralisation of the predictability effect is somehow related to the presence of a constraint manipulation; however, a left-lateralised effect appears to be evenly distributed across previous studies both with and without constraint manipulations. We thus refrain from interpreting the finding, but make note of it as being potentially in need of future characterisation.

### Reflections on Sample Size and the Sequential Bayes Factor Design

A major concern in ERP research is how to balance the labour and financial cost of EEG recordings with statistical power. The sequential Bayes factor design revealed that some research questions may be answerable with relatively small samples. For example, Figure 12 indicates that there was already strong evidence for the standard N400 high versus low cloze probability effect at a sample size of 20 participants. However, here we urge caution: This was a large effect size that had a clear, directional, a priori hypothesis, which we encoded into the statistical model using a truncated prior. A truncated prior will yield strong evidence more quickly for such a large effect, but a truncated prior must be carefully theoretically motivated a priori. Truncated priors will not be suitable for all types of research questions and should be interpreted with a higher threshold for evidence. However, designing informative priors for effects of interest based on previous data may be useful for keeping sample size within practical limits.

Sample size must of course also be large enough to sufficiently account for the effects of interindividual variability and prevalence (i.e., some subjects may be “non-responders”). ERP research is particularly sensitive to interindividual effects given the limitations of the EEG method (i.e., cortical and skull differences, low signal-to-noise ratio), and such effects are difficult to characterise in small samples (we thank an anonymous reviewer for this note). One approach to deciding whether a given sample is sufficiently large when it has been determined via a stopping rule with a narrow, truncated prior is to examine posterior estimates under a range of priors, both truncated and nontruncated, to see how well an estimated effect “holds up” under different assumptions (prior sensitivity analyses should be conducted regardless, but may be additionally useful for this question).

Nonetheless, for our research question regarding constraint, we were able to provide strong evidence of an effect with considerably fewer participants than we had anticipated. For those effects that remained inconclusive at our final sample size, there were reasons we had not anticipated at the design stage of the study (e.g., a pandemic), and we were able to demonstrate using a design analysis that we would not have found strong evidence even with an infeasibly large sample. We were thus able to cut our losses and conserve resources. A sequential Bayes factor design may therefore be an efficient method of sample size determination for future EEG research.

## CONCLUSIONS

In a relatively high-powered experimental design, we confirm previous research demonstrating a dissociated effect of contextual constraint on the ERP, in which the strength of a probabilistic representation affects processing in the post-N400 but not the N400 window. We also demonstrate a dissociated effect of word predictability on the ERP, in which there is a clear effect of predictability in the N400 but not the post-N400 window. Together these findings suggest that N400 amplitude is more sensitive to individual word predictability than context, whereas context is more important than predictability to the processes associated with the post-N400 window. We conclude that in the current study, the processing cost of stronger probabilistic expectations in the post-N400 window resulted from greater conflict between expectations and input, rather than from a greater shift in interpretation or suppression of previous representations. We base this conclusion on our observation of a posterior P600 rather than an anterior PNP. While a shift in interpretation or suppression could have occurred, these processes may not be the inevitable result of strong contextual constraint and may not be mappable to a unique ERP phenomenon. We propose either that eliciting a constraint effect in the anterior PNP requires a more complex experimental design than a straightforward strong/weak constraint comparison or that the constraint-related PNP effect observed in previous studies could even be an artifact of low sample size.

## ACKNOWLEDGMENTS

We thank Johanna Thieke, Romy Leue, and Lisa Plagemann for their help with stimuli development, and for data collection under difficult circumstances during the COVID-19 pandemic. We also thank Trevor Brothers and Gina Kuperberg for sharing data, and Kara Federmeier for sharing stimuli and feedback on the results. Finally, we thank two anonymous reviewers and editor Kate Watkins for their help and feedback at all stages of the review process. Article processing charges were provided by Deutsche Forschungsgemeinschaft (DFG; German Research Foundation) project number 491466077.

## FUNDING INFORMATION

Shravan Vasishth, Volkswagen Foundation, Award ID: 89953. Shravan Vasishth, Deutsche Forschungsgemeinschaft (DFG), Projektnummer: 325493514. Frank Rösler, Deutsche Forschungsgemeinschaft (DFG), Projektnummer: 325493514.

## AUTHOR CONTRIBUTIONS

**Kate Stone**: Conceptualization: Supporting; Formal analysis: Lead; Investigation: Lead; Methodology: Equal; Project administration: Lead; Writing – original draft: Lead; Writing – review & editing: Lead. **Bruno Nicenboim**: Conceptualization: Lead; Formal analysis: Supporting; Methodology: Equal; Project administration: Equal; Supervision: Lead; Writing – review & editing: Supporting. **Shravan Vasishth**: Conceptualization: Equal; Formal analysis: Supporting; Funding acquisition: Equal; Project administration: Equal; Supervision: Equal; Writing – review & editing: Supporting. **Frank Rösler**: Conceptualization: Equal; Formal analysis: Supporting; Funding acquisition: Equal; Project administration: Equal; Supervision: Equal; Writing – review & editing: Supporting.

## SOFTWARE

The following is a complete list of software used for this article: *R* (Version 3.6.3; R Core Team, 2020) and the R-packages bayesplot (Version 1.8.1; Gabry et al., 2019), brms (Version 2.16.3; Bürkner, 2017), eeguana (Version 0.1.8.9001; Nicenboim, 2018), job (Version 0.3.0; Lindeløv, 2021), lme4 (Version 1.1-30; Bates et al., 2015), LSAfun (Version 0.6.3; Günther et al., 2015), patchwork (Version 1.1.1; Pedersen, 2022), rstan (Version 2.21.3; Stan Development Team, 2018, 2020), tidybayes (Version 3.0.2; Kay, 2021), tidyverse (Version 1.3.1; Wickham et al., 2019).
