# Supplementary material for: Understanding the Effects of Constraint and Predictability in ERP
Source: Neurobiol Lang (Camb). 2023 Apr 11;4(2):221–56. doi: 10.1162/nol_a_00094 (PMC10205153; doi:10.1162/nol_a_00094)
Supplement: Supplementary file 1 [file nol-4-2-221-s001.pdf]

Supporting information for the manuscript “Understanding the effects of constraint and predictability in ERP”

Kate Stone[1], Bruno Nicenboim[2], Shravan Vasishth[1], and & Frank Rösler[3]

[1] University of Potsdam

Germany

[2] Tilburg University

Netherlands

[3] University of Hamburg

Germany

Supporting information for the manuscript "Understanding the effects of constraint and predictability in ERP"

Appendix A: List of approved edits to Stage 1 manuscript

During review of the final Stage 2 manuscript, two changes were made to the Stage 1 approved manuscript to improve clarity of the pre-registered hypotheses:

1. The prior specification in paragraph 2 of the *Participants* section was changed to state four priors (one for each hypothesis) rather than the two priors listed in the original Stage 1 pre-registration. While correct, the previous version was not clear about which hypotheses the two priors belonged to.

2. The original bottom right panel of Figure 1 showed a slight positive simulated effect of predictability on the PNP, even though throughout the text we consistently referred to an expected negative effect of predictability on the PNP. The original plot was due to the data simulation, which usually simulated a negative effect, but very occasionally a positive one. The image was therefore replaced with a simulation that yielded a negative effect to better represent our hypotheses.

Appendix B: Cloze probability versus entropy by condition

Table 1 in the main text presents descriptive cloze probability and entropy statistics by condition. Figure 1 below presents these values in a continuous manner, showing the relationship of cloze probability with entropy in each condition.

Figure 1

Log cloze probability per item plotted against entropy in each condition.

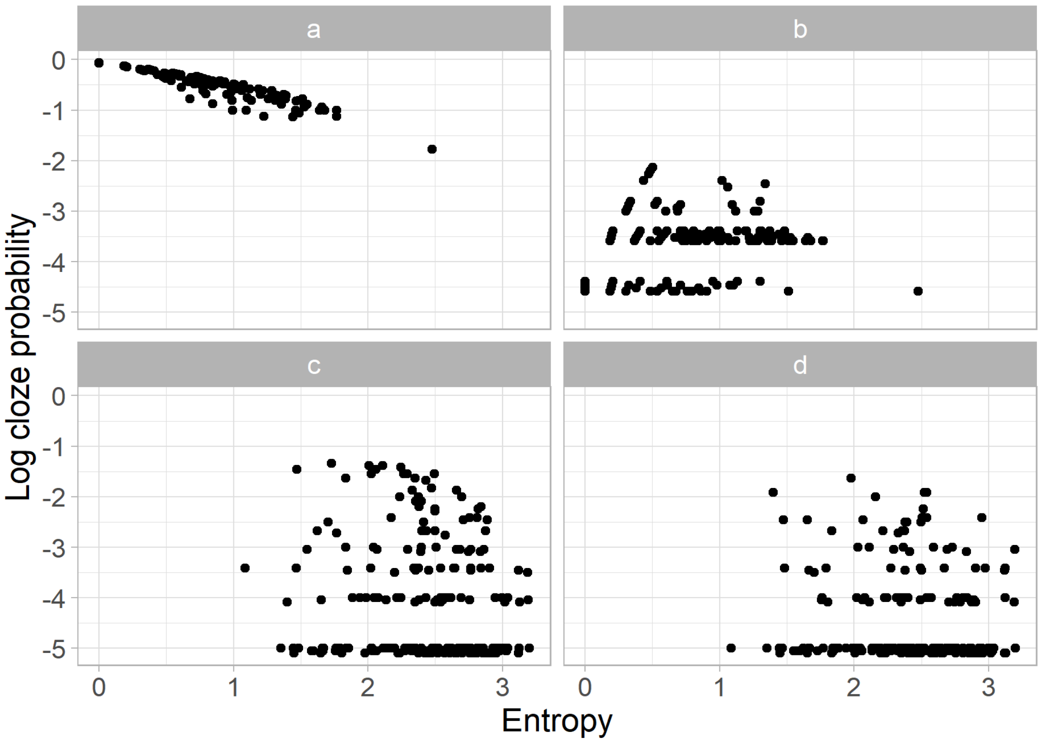

Appendix C: Sensitivity analyses

Sensitivity analyses were conducted to examine how other prior choices may have affected the conclusions of our four pre-registered analyses (Schad et al., 2020). Figure 2A shows the sensitivity analysis for the PNP constraint effect. The analysis indicated that our directional a priori hypothesis of a negative effect of entropy would have yielded strong evidence for the effect at any prior standard deviation of 1 μV or less, with evidence weakening slightly for standard deviations larger than this. A priori non-directional priors would have yielded inconclusive evidence about the effect of entropy on the PNP, but also tended in favour of an entropy effect. Figure 2B suggests that a priori priors assuming a larger range of effect sizes may have yielded moderate to strong evidence against a constraint effect on the N400. We did not analyse truncated priors here as there was no evidence from the literature to support setting a directional prior on the effect of constraint

on the N400, and hence we wanted to rule out any type of effect regardless of the direction. Figure 2C suggests that a priori directional priors assuming even a slightly wider range of larger positive effect sizes would have yielded strong evidence against a predictability effect on the PNP. Non-directional priors would have been less conclusive but tended in the same direction as our pre-registered prior. Finally, Figure 2D suggested consistent conclusions regardless of prior for the standard predictability effect on the N400. In sum, different priors would not have drastically changed our conclusions about any of the effects.

Figure 2

Sensitivity analyses. *The horizontal dashed line at a Bayes factor of 1 in each plot indicates equivocal evidence for H_1 and H_0. Above this line, evidence increases for H_1, below this line, for H_0. Evidence above 10 for H_1 or below 1/10 for H_0 is generally considered to be strong (Jeffreys, 1939). The plot panels show the estimated ratio of evidence for H_1 over H_0 (BF_{10}) using truncated and non-truncated priors with a range of standard deviations for:* ***A.*** *the PNP constraint effect,* ***B.*** *the N400 constraint effect,* ***C.*** *the PNP predictability effect, and* ***D.*** *the N400 predictability effect. The red triangle represents the pre-registered prior used in the main analysis.*

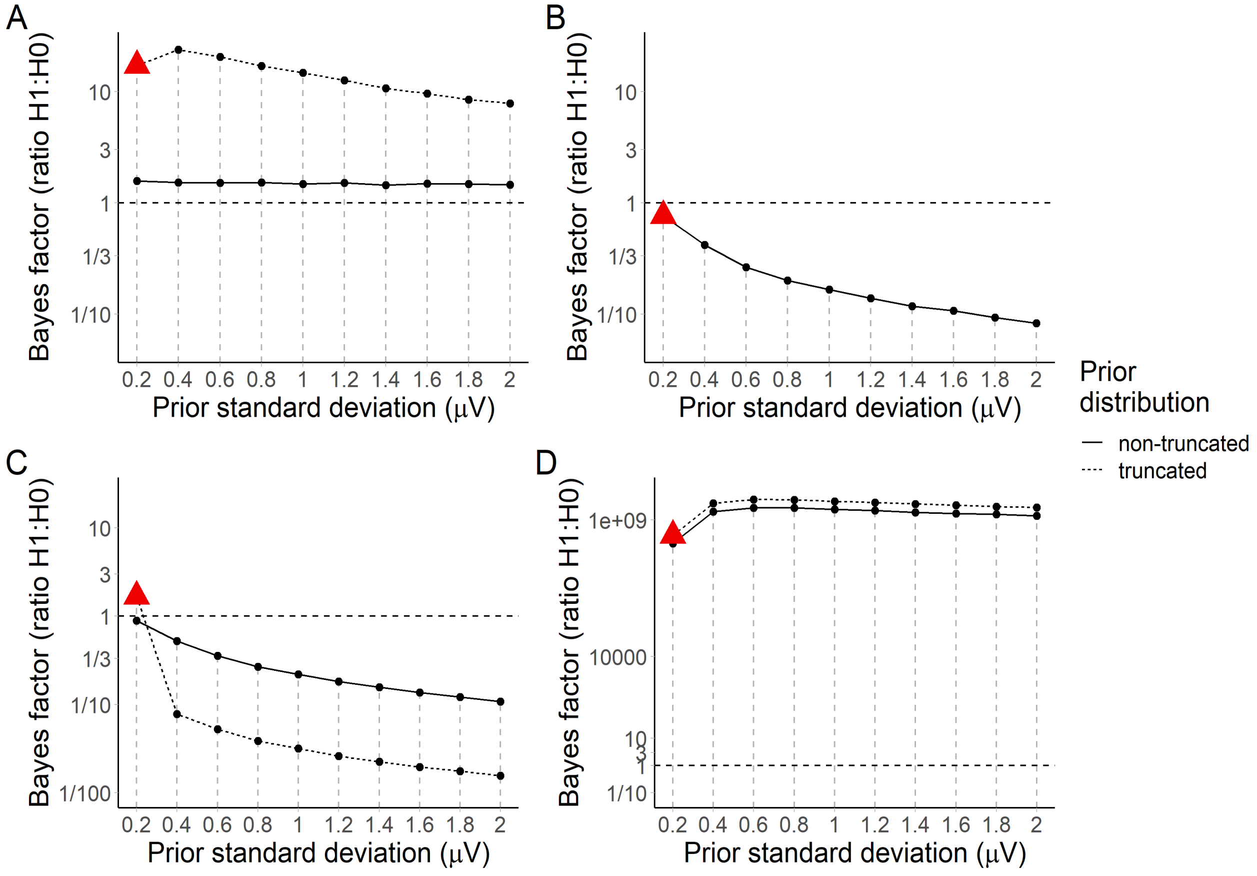

References

Jeffreys, H. (1939). *Theory of Probability*. Oxford University Press.

Schad, D. J., Betancourt, M., & Vasishth, S. (2020). Toward a principled Bayesian workflow: A tutorial for cognitive science. *Psychological Methods*. https://doi.org/10.1037/met0000275
